# Association between electroencephalogram-based sleep characteristics and physical health in the general adult population

**DOI:** 10.1038/s41598-023-47979-9

**Published:** 2023-12-08

**Authors:** Masao Iwagami, Jaehoon Seol, Tetsuro Hiei, Akihiro Tani, Shigeru Chiba, Takashi Kanbayashi, Hideaki Kondo, Takashi Tanaka, Masashi Yanagisawa

**Affiliations:** 1 International Institute for Integrative Sleep Medicine (IIIS), University of Tsukuba, 1-1-1 Tennodai, Tsukuba, Ibaraki 305-8575 Japan; 2 Faculty of Epidemiology and Population Health, London School of Hygiene and Tropical Medicine, Keppel Street, London, WC1E 7HT UK; 3 Department of Health Services Research, Institute of Medicine, University of Tsukuba, 1-1-1 Tennodai, Tsukuba, Ibaraki 305-8575 Japan; 4 Research Center for Overwork-Related Disorders, National Institute of Occupational Safety and Health, Japan (JNIOSH), Kawasaki, Kanagawa 214-8585 Japan; 5 R&D Center for Tailor-Made QOL, University of Tsukuba, Tsukuba, Ibaraki 305-8575 Japan; 6 S’UIMIN Inc., 1-51-1 Hatsudai, Shibuya, Tokyo 151-0061 Japan; 7 Ibaraki Prefectural Medical Center of Psychiatry, 654 Asahimachi, Kasama, Ibaraki 309-1717 Japan; 8 Minamisaitama Hospital, 252 Masumori, Koshigaya, Ibaraki 343-0012 Japan; 9 Department of General Medicine, Institute of Biomedical Sciences, Nagasaki University, 1-12-4 Sakamoto, Nagasaki, 852-8521 Japan; 10 KRD Nihombashi, 4-4-2 Nihonbashi Honcho, Chuo, Tokyo 103-0023 Japan

**Keywords:** Epidemiology, Non-REM sleep, REM sleep, Sleep, Slow-wave sleep

## Abstract

We examined the associations between electroencephalogram (EEG)-based sleep characteristics and physical health parameters in general adults via a cross-sectional study recruiting 100 volunteers aged 30–59 years. Sleep characteristics were measured at home using a portable multichannel electroencephalography recorder. Using the k-means +  +  clustering method, according to 10 EEG-based parameters, participants were grouped into better (n = 39), middle (n = 46), and worse (n = 15) sleep groups. Comparing 50 physical health parameters among the groups, we identified four signals of difference (P < 0.05), including systolic (sBP) and diastolic blood pressure (dBP), γ-glutamyl transpeptidase (γ-GTP), and serum creatinine, where sBP reached a Bonferroni-corrected threshold (P < 0.001). The sBP was higher by 7.9 (95% confidence interval 1.9–13.9) and 15.7 (7.3–24.0) mmHg before adjustment and 5.4 (− 0.1–10.9) and 8.7 (1.1–16.3) mmHg after adjustment for age, sex, body mass index, smoking, drinking habits, and 3% oxygen desaturation index in the middle and worse sleep groups, respectively, than in the better group. As another approach, among 500 combinations of EEG-based and physical health parameters, there were 45 signals of correlation, of which 4 (N1% and sBP, dBP, γ-GTP, and triglycerides) reached a Bonferroni-corrected threshold (P < 0.0001). Thus, EEG-based sleep characteristics are associated with several physical health parameters, particularly sBP.

## Introduction

Accumulating evidence suggests that subjectively and objectively measured sleep parameters are associated with physical and mental health conditions^1–7^. In previous epidemiological studies, self-reported sleeping time and sleep symptoms were associated with the prevalence and incidence of various diseases and mortality^1–4^. Recently, some sleep parameters objectively measured by actigraphy^8–10^, such as sleep duration and irregularity, were associated with subclinical diseases and the prognosis of study participants^5–7^. However, without the electroencephalogram (EEG) function, actigraphy does not provide information about sleep architecture, such as the duration and proportion of rapid eye movement (REM) and non-REM sleep, including stages N1, N2, and N3 (i.e. slow-wave sleep)^11^.

Polysomnography is the gold standard for measuring quantitative sleep parameters. However, extensive time and efforts are required by both examiners and examinees. Therefore, polysomnography is mostly conducted in inpatient settings, targeting unhealthy patients with serious sleeping problems, such as sleep apnea syndrome and narcolepsy. A few studies have conducted polysomnography in home settings in the general population and identified associations between EEG-based sleep parameters and mortality and morbidity, including metabolic syndrome^12–14^. However, these studies were often limited by the dichotomization of disease status (e.g. presence or absence of hypertension and diabetes). Thus, a more detailed measurement of physical health parameters (e.g. blood pressure, HbA1c, and blood lipids), even in the normal range, is warranted to discuss the biological mechanisms connecting sleeping and signs of illness.

A portable multichannel electroencephalography recorder (InSomnograf K2; S’UIMIN Inc., Tokyo, Japan) was recently developed to measure EEG-based sleep parameters of healthy and unhealthy people at home (Supplementary Fig. 1)^15^. Using this modality, we aimed to examine the associations between EEG-based sleep characteristics and 50 physical health parameters (including blood pressure and blood and urine parameters), which can be generally measured at private health check clinics in Japan, in the general population of adults aged 30–59 years via a cross-sectional study. Without particular hypotheses, we used data-driven approaches to (i) identify clusters or groups of people with similar characteristics according to EEG-based sleep parameters and compared the physical health parameters among these groups and (ii) explore which EEG-based sleep parameters and physical health parameters were more strongly correlated than other combinations for the purpose of hypothesis generation.

## Results

### Study participants

Of the 100 study participants aged 30–59 years (mean age 44.0 years, standard deviation [SD] 8.6 years; men, n = 50; women, n = 50), 91, 8, and 1 completed the 5-, 4-, and 3-night sleep measurements using InSomnograf K2, respectively. There were 2, 1, 1, and 1 patients receiving treatment for hypertension, diabetes, hyperuricemia, and dyslipidemia, respectively. In the following analyses, these patients were excluded from the relevant analyses on blood pressure, diabetes, uric acid, and blood lipids because their values were likely to be affected by the drugs.

### Clustering the study participants based on EEG-based sleep parameters

We classified the study participants into groups (“clusters”) according to 10 EEG-based sleep parameters (i.e. total sleep time [TST], sleep efficiency, sleep onset latency, N1%, N2%, N3%, REM%, wake after sleep onset [WASO], arousal index, and sleep stage transitions) using the k-means +  + clustering method^16^. The optimal number of clusters (*k*) was determined to be three, based on the elbow method (Supplementary Fig. 2). Consequently, the study participants were categorized into three groups, including 39, 46, and 15 participants, and were named the “better sleep group,” “middle sleep group,” and “worse sleep group,” respectively, based on the findings shown below.

### Basic characteristics and sleep-related parameters of study participants

The distribution of the basic characteristics and EEG-based sleep parameters is shown in Table 1 and Supplementary Fig. 3. The age distribution was similar between the groups, whereas the middle and worse sleep groups included a higher proportion of men than the better sleep group. Body mass index (BMI) and 3% oxygen desaturation index (ODI) were the highest in the worse sleep group.

**Table 1:** Basic characteristics and sleep parameters of the study participants overall and by electroencephalogram-based sleep cluster.

Variables	Overall (n = 100)	(i) Better sleep group (n = 39)	(ii) Middle sleep group (n = 46)	(iii) Worse sleep group (n = 15)	P value comparing the 3 groups
Age (years), mean ± SD	44.0 ± 8.6	42.0 ± 9.4	45.2 ± 8.2	45.5 ± 7.0	0.1769
Sex (men), n (%)	50 (50.0)	13 (33.3)	27 (58.7)	10 (66.7)	0.0248
Body mass index, mean ± SD	22.2 ± 3.7	21.7 ± 3.6	22.0 ± 2.9	24.0 ± 5.8	0.1243
Smoking habit, n (%)					0.1653
Non-smokers	75 (75.0)	34 (87.2)	32 (69.6)	9 (60.0)	
Past smokers	14 (14.0)	2 (5.1)^a^	9 (19.6)^b^	3 (20.0)^c^	
Current smokers	11 (11.0)	3 (7.7)	5 (10.9)	3 (20.0)	
Drinking habit, n (%)					0.1238
None	29 (29.0)	16 (41.0)	11 (23.9)	2 (13.3)	
≤ 1 day/week	35 (35.0)	12 (30.8)	19 (41.3)	4 (26.7)	
2–5 days/week	19 (19.0)	8 (20.5)	7 (15.2)	4 (26.7)	
≥ 6 days/week	17 (17.0)	3 (7.7)	9 (19.6)	5 (33.3)	
3%ODI (times/hour), mean ± SD	7.3 ± 8.6	5.5 ± 7.4	7.3 ± 8.3	11.9 ± 11.0	0.0500
EEG-based parameters, mean ± SD					
Total sleep time (min)	348.6 ± 58.4	347.6 ± 52.0	348.4 ± 58.5	352.1 ± 75.6	0.9680
Sleep efficiency (%)	90.8 ± 5.3	93.8 ± 3.0	90.2 ± 4.3	84.7 ± 7.2	< 0.0001
Sleep latency (min)	11.2 ± 9.7	7.9 ± 6.7	12.7 ± 9.9	15.4 ± 13.3	0.0142
N1%	8.6 ± 4.8	5.7 ± 2.5	8.9 ± 3.2	15.1 ± 6.6	< 0.0001
N2%	54.2 ± 7.1	49.2 ± 5.5	58.2 ± 5.6	55.3 ± 7.4	< 0.0001
N3%	10.1 ± 7.7	17.0 ± 5.6	4.7 ± 3.9	8.8 ± 7.4	< 0.0001
REM%	26.9 ± 4.9	28.0 ± 4.2	28.2 ± 4.3	20.2 ± 3.0	< 0.0001
WASO (min)	24.4 ± 19.3	14.9 ± 9.5	25.0 ± 16.0	47.1 ± 27.6	< 0.0001
Arousal index (/hour)	11.8 ± 4.6	9.2 ± 3.0	11.4 ± 3.1	19.5 ± 3.6	< 0.0001
Sleep stage transitions (/hour)	7.7 ± 2.2	6.9 ± 1.5	7.3 ± 1.9	10.6 ± 2.2	< 0.0001

*EEG* electroencephalogram, *SD* standard deviation, *ODI* oxygen desaturation index, *REM* rapid eye movement, *WASO* wake after sleep onset.

^a^2 participants quitted smoking ≥ 10 years ago.

^b^6, 1, 1, and 1 participants quitted smoking ≥ 10, 7–9, 4–6, and 1–3 years ago, respectively.

^c^2 and 1 participants quitted smoking 4–6 and 1–3 years ago, respectively.

The average values of most EEG-based sleep parameters were the best in the better sleep group and the worst in the worse sleep group, while the values in the middle sleep group were between those in the better and worse sleep groups. However, TST was not significantly different among the three groups according to the analysis of variance (ANOVA). Among the EEG-based sleep parameters, some were strongly correlated with each other, such as sleep efficiency and WASO (r = − 0.848) and arousal index and sleep stage transitions (r = 0.769) (Supplementary Table 1).

**Figure 1:**
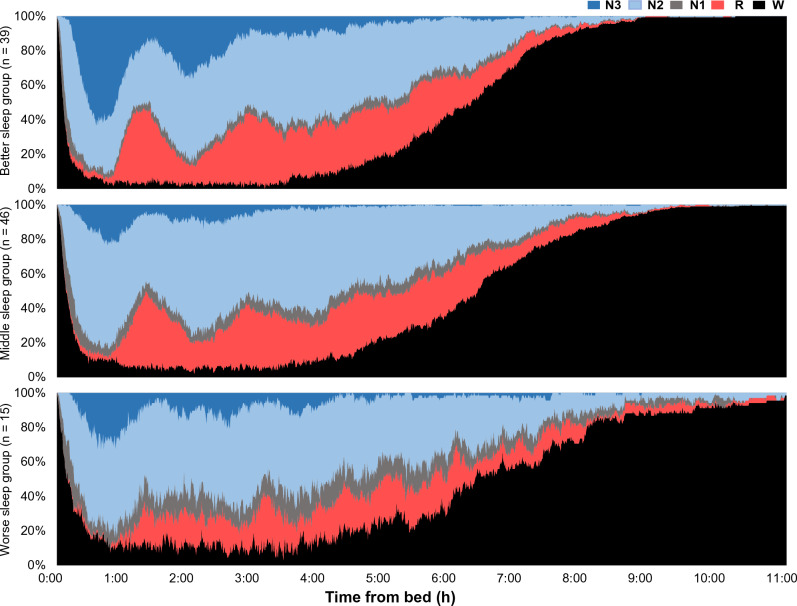
Cumulative displays of sleep architecture by electroencephalogram-based sleep cluster. The X-axis indicates the time from bed, whereas the Y-axis indicates the percentage of people in each sleep stage: awake (“W”), N1, N2, N3, and REM (“R”).

Figure 1 shows the cumulative displays of sleep architecture, illustrating the percentage of people in each sleep stage of awake, N1, N2, N3, and REM (on the Y-axis) according to the time from bed (on the X-axis) by EEG-based sleep cluster. There were apparent differences in sleep architecture, such as the low percentage of N3 in the middle and worse sleep groups and less distinct REM sleep cycles in the worse sleep group.

### Comparison of physical health parameters among the EEG-based sleep clusters

Comparing the 50 physical health parameters among the three groups by ANOVA**,** we identified four signals of difference based on P < 0.05: systolic blood pressure (sBP), diastolic blood pressure (dBP), γ-glutamyl transpeptidase (γ-GTP), and serum creatinine (Supplementary Table 2), of which sBP reached a Bonferroni-corrected significance threshold of P < 0.001 (i.e. 0.05 divided by 50). The mean sBP was the highest in the worse sleep group (119.0 mmHg, SD 14.5 mmHg), followed by that in the middle (111.2 mmHg, SD 14.1 mmHg) and better sleep (103.3 mmHg, SD 13.2 mmHg) groups.

**Figure 2:**
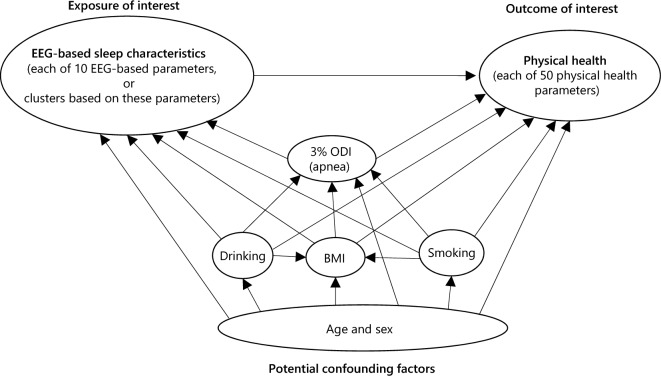
Direct acyclic graph in the association between electroencephalogram-based sleep characteristics and physical health. *BMI* body mass index, *ODI* oxygen desaturation index, *EEG* electroencephalogram.

**Figure 3:**
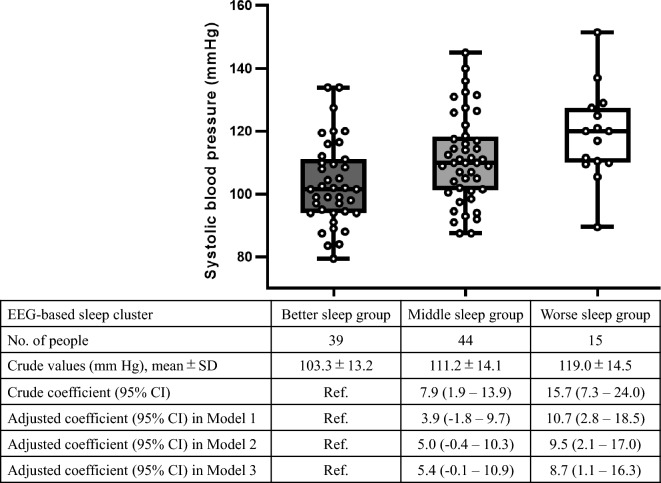
Dot and box plots for systolic blood pressure by electroencephalogram-based sleep cluster and results of univariable and multivariable linear regression analyses. *EEG* electroencephalogram, *CI* confidence interval, *SD* standard deviation. Model 1 is adjusted for age and sex; Model 2 is further adjusted for body mass index, smoking history, and drinking habits; and Model 3 is further adjusted for the 3% oxygen desaturation index. In Model 3, two participants with missing values of 3% oxygen desaturation index are excluded. Each box plot shows the median, interquartile range, and minimum and maximum scores. Two patients receiving treatment for hypertension are excluded.

According to our proposed direct acyclic graph to determine the confounding factors to be adjusted (Fig. 2), we conducted univariable and multivariable regression analyses for sBP (Fig. 3). In the univariable model, sBP was higher by 7.9 (95% confidence interval [CI] 1.9–13.9) and 15.7 (95% CI 7.3–24.0) mmHg in the middle and worse sleep groups, respectively, than that in the better group. In the multivariable model, adjustments for age, sex, BMI, smoking history, drinking habits, and 3% ODI substantially diluted the associations (i.e. β coefficients), suggesting that these factors confounded the association between EEG-based sleep clusters and sBP, in line with the direct acyclic graph. However, there was still an independent association: sBP was higher by 5.4 (95% CI − 0.1–10.9) and 8.7 (95% CI 1.1–16.3) mmHg in the middle and worse sleep groups, respectively, than that in the better group.

The results of multivariable linear regression analyses for other physical health parameters with signals of difference (i.e. dBP, γ-GTP, and serum creatinine) are shown in Supplementary Table 3, suggesting that the associations were diluted to null after adjusting for the aforementioned confounding factors.

### Subgroup analysis by sex

We repeated the analyses according to sex subgroups. The baseline characteristics and results of the multivariable linear regression analyses are shown in Supplementary Tables 4 and 5, respectively. The results in each subgroup were generally similar to those in the main analysis, although the analyses might be underpowered. For example, the average sBP (SD) was 110.2 (12.1), 115.6 (11.7), and 120.7 (16.7) mmHg in men in the better (n = 13), middle (n = 26, excluding 1 patient on medication for hypertension), and worse (n = 10) sleep groups, respectively (P = 0.1602). Meanwhile, it was 99.9 (12.6), 104.9 (15.3), and 115.6 (9.4), in women in the better (n = 26), middle (n = 18, excluding 1 patient on medication for hypertension), and worse (n = 5) sleep groups, respectively (P = 0.0574).

### Subgroup analysis by self-reported Athens insomnia scale (AIS)

Focusing on sBP, we conducted a subgroup analysis using the self-reported AIS, a commonly-used subjective sleep quality scale^17^. Supplementary Fig. 4 shows the distribution of participants according to AIS and EEG-based sleep clusters. The mean AIS score (SD) was 4.9 (3.1), 5.9 (3.5), and 5.9 (3.7) in the better, middle, and worse sleep groups, respectively (ANOVA P-value of 0.435). Only a weak correlation was found between the AIS and EEG-based sleep cluster, with a Spearman’s rank correlation coefficient of 0.123.

**Figure 4:**
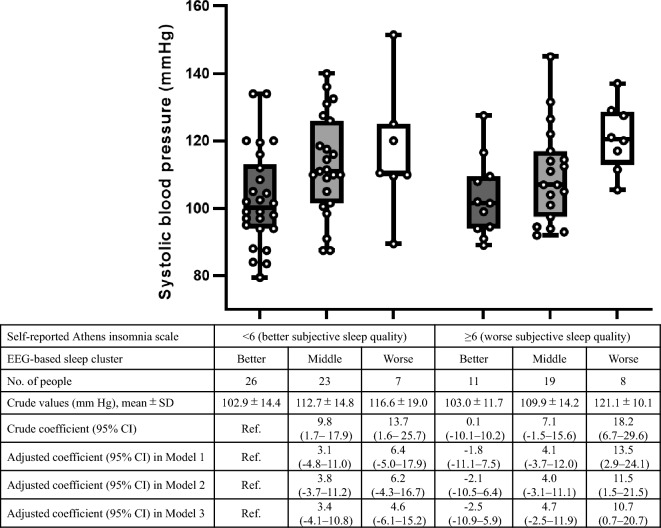
Subgroup analyses for systolic blood pressure according to the Athens insomnia scale. *EEG* electroencephalogram, *CI* confidence interval, *SD* standard deviation. Model 1 is adjusted for age and sex; Model 2 is further adjusted for body mass index, smoking history, and drinking habits; and Model 3 is further adjusted for the 3% oxygen desaturation index. In Model 3, two participants with missing values of 3% oxygen desaturation index are excluded. Each box plot shows the median, interquartile range, and minimum and maximum scores. Two patients receiving treatment for hypertension and four patients with missing values of Athens insomnia scale are excluded.

In the subgroup with AIS scores < 6 and ≥ 6 (a commonly used cut-off to define insomnia^17^), the association with sBP was more prominent for the EEG-based sleep clusters than that for the AIS (Fig. 4). Putting the AIS and EEG-based sleep clusters together in the multivariable linear regression model (adjusted for age, sex, BMI, smoking history, and drinking habits, and 3% ODI), there was almost no association for AIS (adjusted β coefficient per 1 increase of AIS 0.04 [95% CI − 0.7–0.8]), whereas sBP was higher by 4.9 (95% CI − 0.7–10.5) and 8.7 (95% CI 0.9–16.6) mmHg in the middle and worse sleep groups, respectively, than that in the better group.

### Sensitivity analyses

Sensitivity analyses (i) excluding one patient (in the middle sleep group) using sleeping medication and (ii) adjusting for the average 3% ODI of two nights (in the fully-adjusted model) instead of the worse value in the main analysis (where the correlation between the average and worse values was very high [r = 0.979]) showed almost the same results to the main analysis, including the results for sBP (Supplementary Table 6).

### Correlations between each EEG-based sleep parameter and physical health parameter

As another approach with a highly exploratory purpose, we comprehensively estimated the correlation coefficients of all 500 combinations between the 10 EEG-based sleep parameters and 50 physical health parameters. In total, 45 signals of correlations were identified based on P < 0.05 (Supplementary Table 7), of which 4 combinations reached a Bonferroni-corrected significance threshold of P < 0.0001 (i.e. 0.05 divided by 500): N1% and sBP, dBP, γ-GTP, and triglycerides. In the linear regression models for these combinations, adjustment for age, sex, BMI, smoking history, drinking habits, and 3% ODI diluted the associations to the null for sBP and dBP. In contrast, independent associations were found for γ-GTP and triglycerides, with adjusted β coefficients of 6.4 (95% CI 0.8–11.9) IU/L and 13.7 (95% CI 3.4–24.1) mg/dL, respectively, per 1 SD increase of N1%.

The results of multivariable linear regression analyses for all combinations with signals of correlations (P < 0.05) are shown in Supplementary Table 8. Most associations were diluted to the null after adjusting for the aforementioned confounding factors, while some independent associations (as indicated by 95% CIs of adjusted β coefficients not overlapping with zero) were found: (i) TST and total homocysteine, (ii) sleep efficiency and fasting blood glucose, (iii) sleep efficiency and zinc, (iv) sleep efficiency and blood urea nitrogen (BUN), (v) WASO and fasting blood glucose, (vi) WASO and HbA1c, and (vii) sleep stage transition and ferritin levels.

## Discussion

We comprehensively examined the associations between EEG-based sleep parameters and physical health in the general adult population. The key findings are as follows. Among the 50 physical health parameters, there were four signals of difference (sBP, dBP, γ-GTP, and serum creatinine) in the EEG-based sleep clusters, of which sBP reached a Bonferroni-corrected threshold. After adjusting for potential confounding factors, the EEG-based sleep clusters were independently associated with sBP. In addition, subjective (AIS) and objective (EEG-based) sleep qualities were often discrepant, and sBP was associated with EEG-based sleep clusters but not with AIS. In another data-driven analysis, among 500 combinations between each EEG-based sleep parameter and each physical health parameter, we identified 45 signals of correlations, of which 4 (N1% and sBP, dBP, γ-GTP, and triglycerides) reached a Bonferroni-corrected significance threshold. Notably, sleep quantity (TST) was similar among the EEG-based sleep clusters and was not correlated with most physical health parameters. The current study highlights the importance of objective sleep quality rather than sleep quantity, as well as subjective sleep quality.

To the best of our knowledge, this is the first study to measure EEG-based sleep parameters at home in the general adult population using InSomnograf K2, a newly developed portable multichannel electroencephalography recorder. The novelty of the present study includes our application of clustering approach to sleep evaluation. The study participants were classified into three groups using the k-means +  + clustering method. We believe that clustering, one of the unsupervised machine learning methods^18^, is a reasonable approach because of the following reasons. First, there are many EEG-based sleep parameters that are correlated with each other, such that no single parameter should not be used to classify people. Second, there is no universally established criteria to differentiate “good” and “bad” sleep based on EEG-based sleep parameters. We believe that our clustering was successful, because most EEG-based sleep parameters were significantly different among the three groups.

We found significant differences in sBP among the EEG-based clusters. The sBP was higher by 7.9 mmHg and 15.7 mmHg before adjustment and by 5.4 mmHg and 8.7 mmHg after adjustment for age, sex, BMI, smoking history, drinking habits, and 3% ODI in the middle and worse sleep groups, respectively, than in the better sleep group. The considerable decrease in the estimates after the adjustment indicate that these factors indeed served as “confounding factors,” as we expected with our proposed direct acyclic graph. For example, obesity and drinking are likely to affect both EEG-based sleep parameters and some physical health parameters. However, even after adjustment of these factors, there was an independent association between the EEG-based clusters and sBP. This fact may suggest that, regardless of obesity and drinking, sleep quality itself affects sBP. For example, poor sleep quality could damage the balance between the sympathetic and parasympathetic nerves and stress the blood vessels, increasing blood pressure^19^.

Previous studies examined the association between EEG-based sleep parameters and blood pressure. In a cross-sectional study of 62 normal adults without sleep apnea, focusing on nocturnal blood pressure dipping, deeper and less-fragmented sleep was associated with more blood pressure dipping^20^. In another study, 11 healthy adults were selectively deprived of slow-wave sleep, leading to significantly attenuated mean arterial bloop pressure dipping during the first half of total sleep^21^. In the Study of Women's Health Across the Nation Sleep Study, the EEG total beta power during non-REM sleep was positively associated with hypertensive status, whereas sleep duration and efficiency were not associated with blood pressure cross-sectionally or longitudinally^22^. These studies, as well as the present study, suggest that sleep quality measured by EEG affects blood pressure.

The participants, only two of whom were receiving treatment for hypertension, in the current study were even healthier than those in previous studies targeting the general population^12, 13^. However, it is noteworthy that EEG-based sleep clusters showed this level of difference in sBP among adults aged 30–59 years. If their sleep quality remains unchanged, the difference in sBP between the groups may become higher with aging, eventually leading to cardiovascular events (e.g. stroke and myocardial infarction) in the worse sleep group.

In addition, the AIS, a subjective measure of sleep quality, was not strongly correlated with EEG-based sleep clusters. This finding aligns with previous studies suggesting that EEG-based sleep quality is not reliably predictive of self-reported sleep quality in healthy adults^23, 24^. Importantly, there was almost no association between AIS and sBP, whereas the EEG-based clusters were still independently associated with sBP after co-adjustment of AIS scores. From a research perspective, the current study highlights the importance of objective, rather than subjective, measures of sleep quality in epidemiological studies on the association between sleep and physical health. From a clinical perspective, our findings indicate that it would be ideal to measure EEG-based sleep quality, instead of self-reported sleep quality.

This study also provides hypotheses for further research on the association between EEG-based sleep characteristics and physical health. Correlation analysis suggested that, among the 10 EEG-based sleep parameters, N1% showed a relatively strong correlation with blood pressure, γ-GTP, and triglycerides. Furthermore, despite multiple testing, an independent association was observed for (i) TST and total homocysteine, (ii) sleep efficiency and fasting blood glucose, (iii) sleep efficiency and zinc, (iv) sleep efficiency and BUN, (v) WASO and fasting blood glucose, (vi) WASO and HbA1c, and (vii) sleep stage transition and ferritin levels. These findings suggest that certain EEG-based sleep characteristics are not only associated with blood pressure, but also with metabolic functions and inflammation, in line with previous results^25–27^. Both sleep duration and efficiency mediate the connection between homocysteine levels and oxidative stress^25^. One possibility for this is that frequent and repeated WASO might lead to increased concentrations of ferritin levels due to oxidative stress^28^.

The current study has some limitations. First, this was a cross-sectional study, and thus, a temporal relationship between EEG-based sleep parameters and physical health parameters could not be established. Based on previous studies suggesting that sleep quality was associated with the future incidence of metabolic disorders^5, 7, 13, 29^, we assumed that sleep quality affected physical health. However, the opposite may also be true, that is, physical conditions may also affect sleep quality^30^. Prospective cohort studies or Mendelian randomization studies (an instrumental variable analysis regarding genetic variants as instrumental variables) are warranted to examine any causal relationship and direction of causation, especially by focusing on the combinations with the strong correlation suggested in the current study. Second, misclassification of the studied parameters is possible. EEG-based parameters were measured by InSomnograf K2, which may be less accurate than those measured with the gold-standard method of polysomnography^15^. Blood samples were measured in the morning, although some parameters (e.g. blood cell counts) could change within a 24-h period.

Third, the sample size in this study was not very large. We were unable to calculate the necessary or sufficient sample size in advance because this was an exploratory, data-driven study without any particular hypothesis. The number of participants per group was automatically determined in the clustering analysis. Consequently, the worse sleep group included a relatively small number of participants, limiting the power of statistical analysis. We conducted Bonferroni corrections for our multiple testing in the crude comparison and found “statistically significant” associations or correlations. However, the study did not have enough statistical power to conclude that the adjusted associations in the multivariable analyses were “statistically significant” if multiple tests were considered. In addition, in our clustering analysis, we had to set the number of clusters to be three because four or more clusters would include a small number of people and limit meaningful comparisons. A clustering analysis with a relatively larger sample size may identify a cluster of people with more specific sleep characteristics. Further studies with relatively larger sample sizes, including unhealthier people, are warranted to test whether these hypotheses are true or only a chance finding. Finally, although we collected and adjusted for potential confounding factors in the association between the EEG-based sleep clusters and sBP according to our proposal of direct acyclic graph, residual confounding is still possible due to rough categorization and potential misclassification of the variables. Therefore, the independent association we detected may be partly explained by the residual confounding.

## Conclusions

In this cross-sectional study measuring EEG-based sleep parameters at home, objectively measured sleep quality, rather than sleep quantity and subjectively measured sleep quality, was associated with several aspects of physical health, particularly sBP, in the general population of adults aged 30–59 years. This study highlights the importance of measuring EEG-based sleep quality in clinical practice and epidemiological studies. Further prospective cohort studies are warranted to examine whether these sleep characteristics are associated with long-term disease incidence and prognosis and, ultimately, whether modifying objectively measured sleep quality could prevent diseases.

## Methods

### Study design, setting, and participants

In this cross-sectional observational study, we recruited study participants from a private health check clinic in Tokyo, Japan between October and December 2021. The clinic offers comprehensive medical checkups as a private service. For the current study, we recruited voluntary participants aged 30–59 years, on-site and from the Internet, who agreed to receive comprehensive medical checkups and measurement of EEG-based sleep parameters using InSomnograf K2. To ensure a balanced age-sex distribution, we recruited up to 17 participants from each sex and 10-year age stratum from 30 to 59 years, in a total of 100 individuals. We did not set any exclusion criteria for study participants to represent the general population. The participants were not paid, but they could receive the comprehensive medical checkup for free. We were unable to conduct any sample size calculation because this was an exploratory study without particular hypotheses and also because we could not predict the number of people in each group in the clustering analysis. Thus, the sample size of 100 was determined realistically with respect to time and cost.

### Ethical approval

This study was conducted according to the principles of the Declaration of Helsinki. The study was approved by the Institutional Review Board of Daichinokai Medical Corporation. Informed consent was obtained from all study participants.

### Measurements of sleep characteristics

Details of the InSomnograf K2 are described in a previous study using this modality for older people^15^. In brief, InSomnograf K2 is a newly developed modality to measure brain waves during sleep, with five soft sticking electrodes placed on the head in the frontal and occipital locations and connected to a portable recording machine (Supplementary Fig. 1). The modality was validated against a typical polysomnography device with a concordance rate of 86.9% and kappa coefficient of 0.80^15^. In addition, there was a high correlation between InSomnograf K2 and self-reported questionnaires on bedtime (r = 0.935), waking time (r = 0.933), and midpoint of sleep time (r = 0.967)^15^.

The recording system consisted of five electroencephalogram derivations (Fp1–M2, Fp2–M1, Fp1–average M, Fp2–average M, and Fp1–Fp2). The recorded nights were divided into 30 sequential periods and manually classified into REM sleep and non-REM sleep, which were further classified into light sleep (N1 and N2, separately) and slow-wave sleep (N3).

Using this modality, in line with the sleep quality recommendations of the National Sleep Foundation^11^, we listed and measured the following sleep parameters for five nights: TST; sleep efficiency (%), defined as dividing TST by total bedtime; sleep onset latency (min), defined as the duration from the time in bed to the sleep onset; percentage of stage N1, N2, N3, and REM, dividing the duration of each by TST; WASO (min), defined as the amount of time spent awake after sleep had been initiated and before final awakening; and arousal index (/hour), defined as the average number of arousals (an abrupt change from “deeper” stage of non-REM sleep to a “lighter” stage or from REM sleep toward wakefulness) per hour of sleep^11^. The scores were determined according to the American Academy of Sleep Medicine criteria^31^. In addition, we measured sleep stage transitions (/hour), defined as the frequency of transitions among waking, non-REM sleep, and REM sleep, as they were associated with self-reported restlessness and light sleep independent of the arousal index^32^.

For these 10 parameters, the average values from the 5 nights were calculated and used in the following analyses. Most participants completed the measurements on five nights, but some failed on one or more nights. For these participants, we took the average of the sleeping parameters for all available nights. During the five nights, the study participants were asked to maintain their usual/regular activities, including drinking and sports activities.

In addition, the study participants were asked to fill in the electronic form that included questions to calculate the AIS score. Briefly, the AIS is a validated self-assessment psychometric instrument designed for quantifying sleep difficulty based on the International Classification of Disease-10 criteria. It consists of eight items: sleep induction, awakenings during the night, final awakening, total sleep duration, sleep quality, well-being, functioning capacity, and sleepiness during the day^17^. Each item is scored from 0 to 3, and the total score ranges from 0 (denoting absence of any sleep difficulty) to 24 (denoting most severe sleep difficulty). The electronic form also included questions on smoking and drinking habits and use of medications for sleeping and other conditions such as hypertension and diabetes.

The 3% ODI, defined as the number of oxygen desaturations ≥ 3% per hour of sleep^33, 34^, was measured using a portable pulse oximeter (RingO2; Neuroceuticals Inc., Tokyo, Japan) for two nights, and the higher (i.e. worse) value of the two nights was selected.

### Clustering the study participants based on EEG-based sleep parameters

Using the k-means +  + clustering method, we classified the study participants into several groups (“clusters”) of people with similar characteristics, according to 10 EEG-based parameters, namely, TST, sleep efficiency, sleep onset latency, N1%, N2%, N3%, REM%, WASO, arousal index, and sleep stage transitions. All of these were standardized as mean 0 and standard deviation 1. K-means clustering is a method of vector quantization that aims to partition the observations or study participants into *k* (i.e. the number of clusters) clusters, in which each observation belongs to the cluster with the nearest mean. K-means +  + is a modified version of the k-means method, with an algorithm for choosing the initial values before proceeding with the standard k-means optimization iterations for the results of clustering to be more stable than the original k-means method^16^. The optimal number of clusters (*k*) was determined based on the elbow method showing the “distortion” according to the number of clusters, as well as the number of people in each cluster to avoid too small clusters. After the clustering analysis, we interpreted the characteristics of each group and named each group if possible.

### Measurements of physical health parameters

Physical health parameters were measured in the morning (around 8–10 AM), without breakfast except for a cup of water, with the participants requested to have the last meal before 9 PM on the day before measurement. Blood pressure measurements were conducted twice using an automated machine (MPV-5500; NIHON KOHDEN Co., Tokyo, Japan) and averaged. Blood and urine samples were collected and measured at a central laboratory affiliated with the study site. The measured hematological parameters included fasting blood glucose, hemoglobin A1c (national glycohemoglobin standardization program); glycoalbumin; 1,5-anhydro-D-glucitol; hemoglobin; white blood cell count; platelet count; total iron-binding capacity; unsaturated iron-binding capacity; ferritin; serum iron; serum copper; C-reactive protein (CRP); ceruloplasmin; zinc; 25-hydroxyvitamin D; 1,25-dihydroxyvitamin D; calcium; magnesium; sodium; potassium; chloride; uric acid; total protein; albumin; aspartate aminotransferase; alanine transaminase; γ-GTP; lactate dehydrogenase; alkaline phosphatase; total, direct, and indirect bilirubin; serum amylase; choline esterase; creatinine; BUN; total cholesterol; triglyceride; high-density lipoprotein cholesterol; low-density lipoprotein (LDL) cholesterol; small dense LDL cholesterol; phospholipid; total homocysteine; free fatty acids; serum pepsinogen I; and serum pepsinogen I/II ratio. Urine measurements were conducted for urine albumin per 1 g of creatinine. In total, 50 physical health parameters were assessed. Red blood cell count and hematocrit were excluded because they were largely correlated with hemoglobin levels (r = 0.837 and 0.974, respectively).

### Statistical analysis

First, we described the basic characteristics, EEG-based sleep parameters, and physical health parameters of the overall study participants and by EEG-based sleep clusters. The three groups were compared using ANOVA for continuous variables and χ^2^ tests for sex, smoking history, and drinking habits. In addition, the correlation coefficients among the EEG-based sleep parameters were estimated. All sleep and physical parameters in the current study were continuous; therefore, they were summarized as the mean ± SD. The parameters suggestive of log-normal distribution, including CRP and urine albumin, were log-transformed with a base of 10.

In each group, we illustrated the cumulative displays of sleep architecture, showing the percentage of people in each sleep stage of awake, N1, N2, N3, and REM (on the Y-axis) according to the time from bed (on the X-axis)^15^.

Then, we crudely compared the physical health parameters among the three groups using ANOVA. Signals of difference were identified based on P < 0.05 and a Bonferroni-corrected statistical significance threshold of P < 0.001 (i.e. 0.05 divided by 50). The parameters with signals of difference were included in the univariable and multivariable linear regression analyses in which the exposure was the EEG-based sleep cluster (with reference to the better sleep group) and the outcome was each physical health parameter. According to our proposed direct acyclic graph to determine confounding factors to be adjusted (Fig. 2), we adjusted for age (as a continuous variable) and sex (Model 1); further for BMI (as a continuous variable), smoking history (non-smokers, past smokers, and current smokers), and drinking habits (none, ≤ 1 day/week, 2–5 days/week, and ≥ 6 days/week) (Model 2); and further for 3% ODI (as a continuous variable) (Model 3) because sleep apnea was a well-known risk factor for impaired physical health^35, 36^. We also conducted a subgroup analysis by sex.

In addition, the AIS scores were compared among the clusters, and the Spearman’s correlation coefficient between the AIS and EEG-based clusters was estimated. Then, for the subgroup analysis, we differentiated participants with AIS scores < 6 and ≥ 6 and conducted univariable and multivariable linear regression analyses to compare the six groups according to the AIS and EEG-based sleep clusters. Further, we included both the AIS (as a continuous variable) and EEG-based sleep clusters in the multivariable linear regression model.

Sensitivity analyses (i) excluding one patient using sleep medication and (ii) adjusting for the average 3% ODI of two nights (in the fully-adjusted model) instead of the worse value were also conducted.

As another data-driven approach, we comprehensively estimated the correlation coefficients of all the combinations between the 10 EEG-based parameters and 50 physical health parameters to identify signals of correlation based on P < 0.05 and a Bonferroni-corrected statistical threshold of P < 0.0001 (i.e. 0.05 divided by 500). For combinations with signals of correlation, univariable and multivariable linear regression analyses in which the exposure was the EEG-based sleep parameter (per 1 SD increase) and the outcome was the physical health parameter were also conducted.

We used STATA version 17 for the statistical tests, Python version 3.7.7 (“sklearn” package) to conduct the K-means +  + clustering, Microsoft Excel version 2021 to illustrate Fig. 1, and GraphPad Prism 9 to illustrate Figs. 3 and 4 and Supplementary Fig. 3.

### Supplementary Information

Supplementary Information.

## Data Availability

The data used in this study were licensed by S’UIMIN Inc. The data have not been made publicly available and could be used in future projects to develop medical devices and diagnostic technologies. Proposals and requests for data access should be directed to the corresponding author via email.
