# Supplementary material for: Association between electroencephalogram-based sleep characteristics and physical health in the general adult population
Source: Sci Rep. 2023 Dec 8;13:21545. doi: 10.1038/s41598-023-47979-9 (PMC10709300; doi:10.1038/s41598-023-47979-9)
Supplement: Supplementary file 1 — Supplementary Information. [file 41598_2023_47979_MOESM1_ESM.docx]

**Supplementary Figure 1. Image of InSomnograf K2**

**
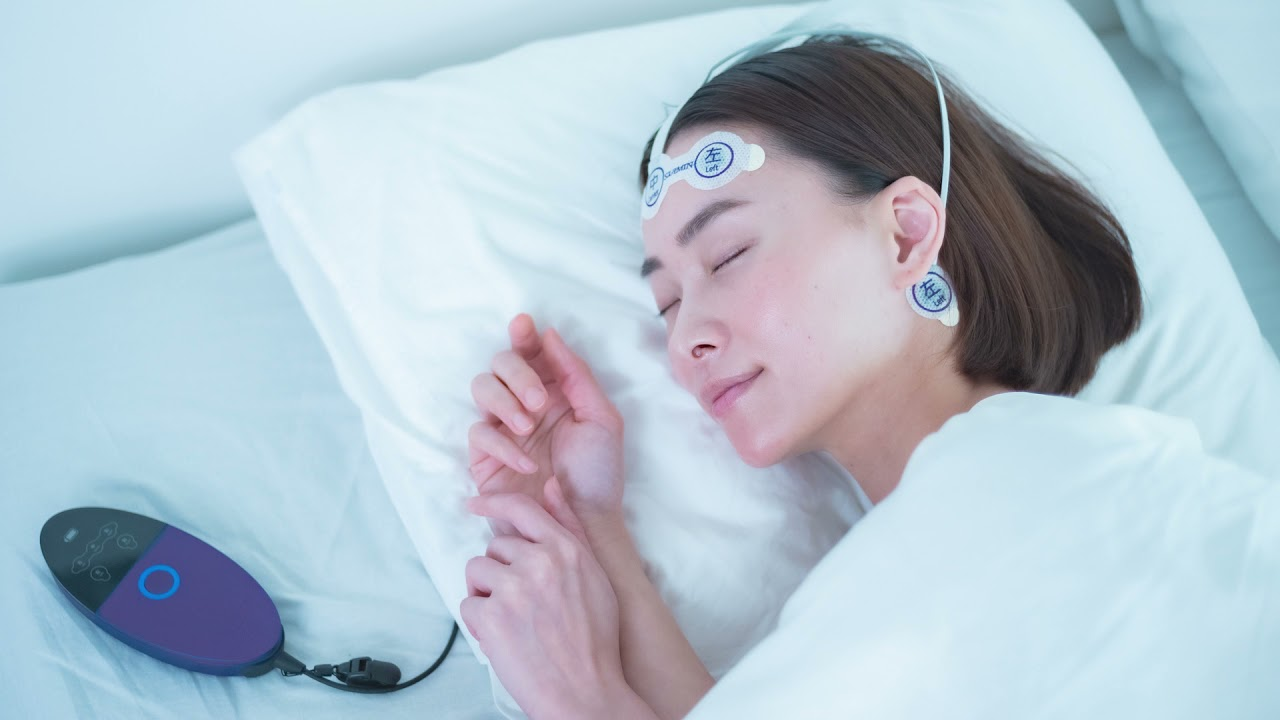
**

Publicly available at https://www.suimin.co.jp/

**Supplementary Figure 2. Result of elbow method in the k-means++ clustering method**


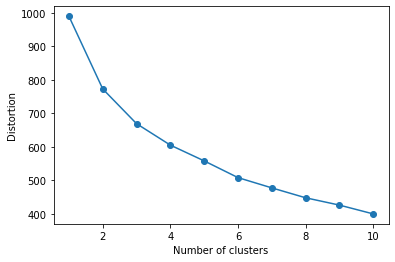


*Commands used in the Python version 3.7.7:

from sklearn.cluster import KMeans

distortions = []

for i in range(1,11):

km = KMeans(n_clusters=i, init='k-means++', n_init=10, max_iter=300, random_state=0)

km.fit(dataset)

distortions.append(km.inertia_)

plt.plot(range(1,11),distortions,marker='o')

plt.xlabel('Number of clusters')

plt.ylabel('Distortion')

plt.show()

**
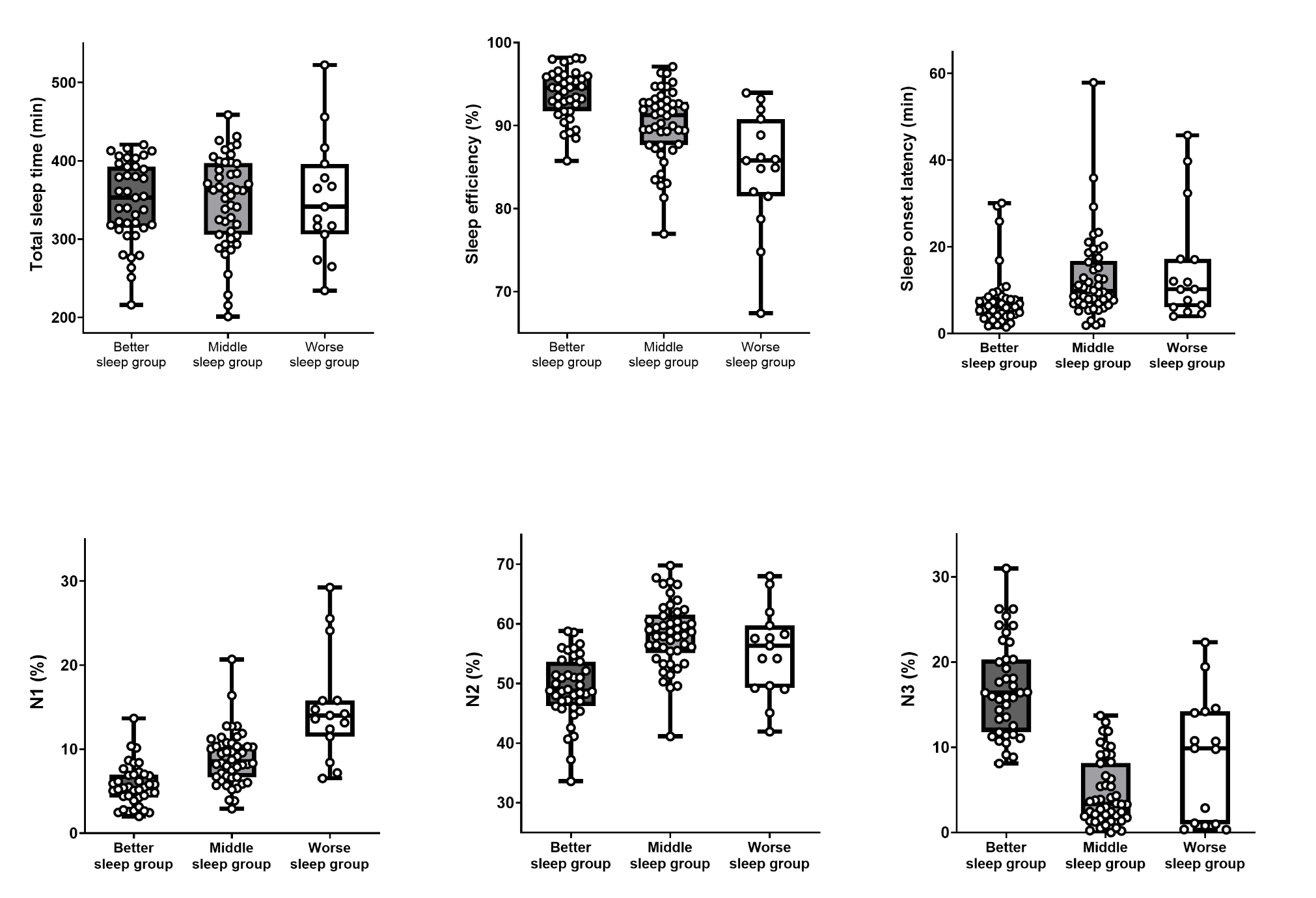
Supplementary Figure 3. Box plots of electroencephalogram-based sleep parameters by electroencephalogram-based cluster**


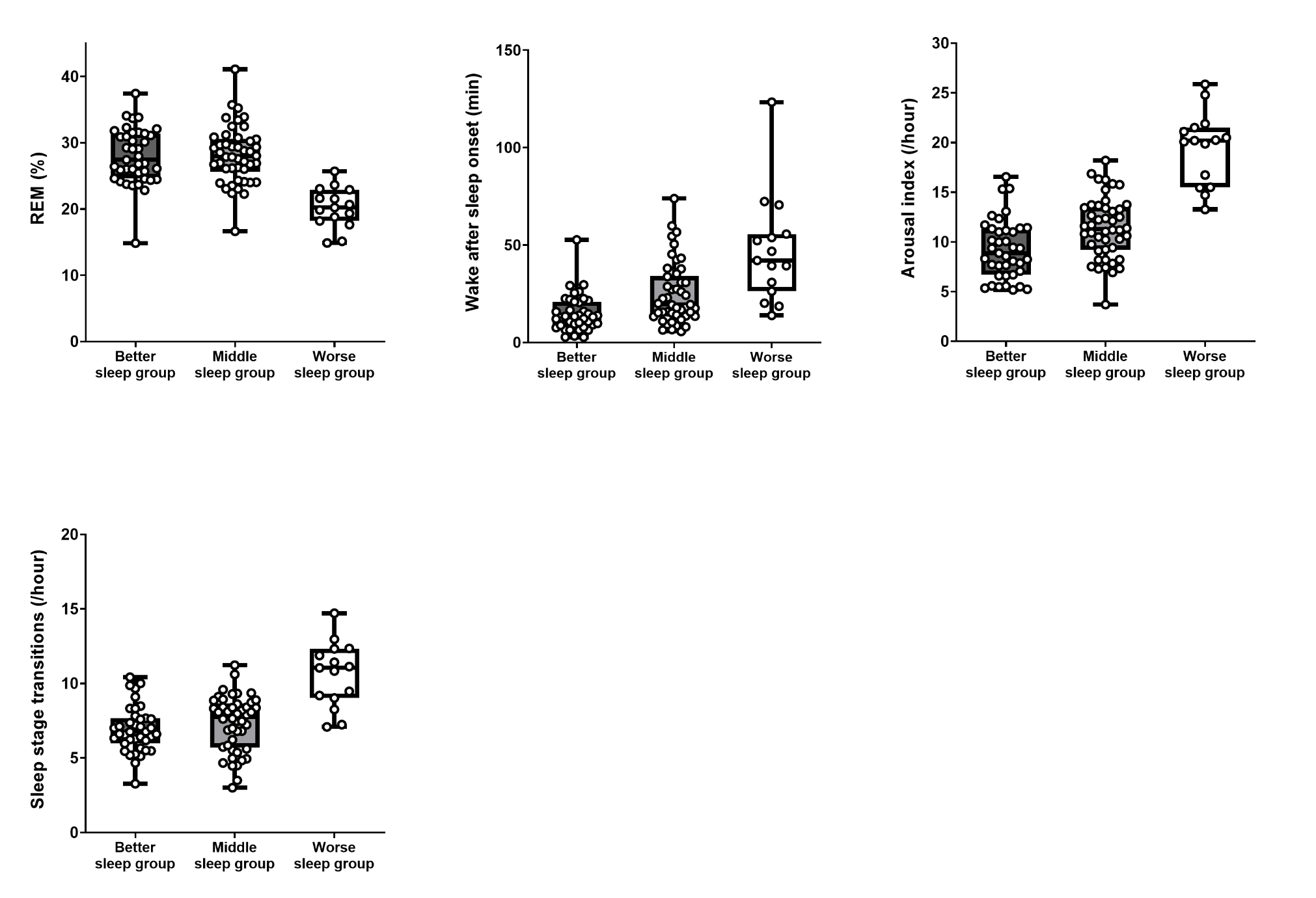
 * Each box plot shows the scores of median, interquartile range, minimum, and maximum.

**Supplementary Table 1. Correlation coefficients among electroencephalogram-based sleep parameters**

|  | Total sleep time | Sleep efficiency | Sleep onset latency | N1％ | N2% | N3% | REM% | WASO | Arousal index | Sleep stage transitions |
| --- | --- | --- | --- | --- | --- | --- | --- | --- | --- | --- |
| Total sleep time | 1.000 | - | - | - | - | - | - | - | - | - |
| Sleep efficiency | 0.162 | 1.000 | - | - | - | - | - | - | - | - |
| Sleep onset latency | 0.053 | -0.583 | 1.000 | - | - | - | - | - | - | - |
| N1% | 0.133 | -0.379 | 0.229 | 1.000 | - | - | - | - | - | - |
| N2% | -0.155 | -0.190 | 0.045 | -0.029 | 1.000 | - | - | - | - | - |
| N3% | 0.024 | 0.258 | -0.209 | -0.445 | -0.677 | 1.000 | - | - | - | - |
| REM% | 0.044 | 0.256 | 0.020 | -0.289 | -0.339 | -0.136 | 1.000 | - | - | - |
| WASO | 0.115 | -0.848 | 0.207 | 0.382 | 0.155 | -0.187 | -0.320 | 1.000 | - | - |
| Arousal index | 0.062 | -0.367 | 0.097 | 0.693 | -0.019 | -0.178 | -0.414 | 0.385 | 1.000 | - |
| Sleep stage transitions | -0.049 | -0.166 | -0.094 | 0.534 | -0.042 | -0.042 | -0.422 | 0.196 | 0.769 | 1.000 |

REM = rapid eye movement, WASO = wake after sleep onset.

**Supplementary Table 2. Physical health parameters of the study participants overall and by electroencephalogram-based sleep cluster**

| No. | Variables, mean±SD | Overall  (n=100) | (i) Better sleep group  (n=39) | (ii) Middle sleep group  (n=46) | (iii) Worse sleep group  (n=15) | P value comparing 3 groups by ANOVA |
| --- | --- | --- | --- | --- | --- | --- |
| **1** | **Systolic blood pressure (mmHg)^a^** | **109.2±14.8** | **103.3±13.2** | **111.2±14.1** | **119.0±14.5** | **0.0008** |
| **2** | **Diastolic blood pressure (mmHg)^a^** | **67.8±11.3** | **63.6±9.9** | **69.5±11.1** | **73.9±11.7** | **0.0035** |
| 3 | Fasting blood glucose (mg/dL)^b^ | 95.3±9.1 | 93.3±8.7 | 95.8±6.5 | 99.3±14.7 | 0.0871 |
| 4 | HbA1c (National Glycohemoglobin Standardization Program) (%)^b^ | 5.3±0.3 | 5.3±0.2 | 5.3±0.3 | 5.4±0.5 | 0.3961 |
| 5 | Glycoalbumin (%)^b^ | 14.1±1.4 | 14.2±1.4 | 14.2±1.4 | 13.6±1.3 | 0.3766 |
| 6 | 1,5-anhydro-D-glucitol (μg/mL)^b^ | 19.5±6.9 | 19.6±6.6 | 19.2±6.7 | 20.0±8.8 | 0.9121 |
| 7 | Hemoglobin (g/dL) | 14.3±1.4 | 13.9±1.4 | 14.5±1.5 | 14.5±1.1 | 0.1417 |
| 8 | White blood cell count (/μL) | 5098±1405 | 5038±1665 | 5180±1069 | 5000±1649 | 0.8626 |
| 9 | Platelet count (10^4^/μL) | 24.8±5.5 | 24.7±5.6 | 24.7±5.0 | 25.5±6.8 | 0.8754 |
| 10 | Total iron binding capacity (μg/dL) | 318.5±45.1 | 316.0±40.1 | 318.6±48.7 | 324.9±48.5 | 0.8153 |
| 11 | Unsaturated iron binding capacity (μg/dL) | 207.5±61.6 | 207.5±57.2 | 203.4±67.9 | 220.2±54.0 | 0.6609 |
| 12 | Ferritin (ng/mL) | 106.7±96.1 | 86.0±79.1 | 116.2±98.7 | 131.5±121.6 | 0.1977 |
| 13 | Serum iron (μg/dL) | 111.0±36.4 | 108.5±34.3 | 115.2±39.4 | 104.7±32.4 | 0.5429 |
| 14 | Serum copper (μg/dL) | 100.8±23.2 | 98.8±18.7 | 102.0±27.3 | 102.7±21.0 | 0.7739 |
| 15 | C-reactive protein (mg/dL) (common log-transformed) | -1.38±0.48 | -1.49±0.42 | -1.37±0.43 | -1.17±0.71 | 0.0857 |
|  | (C-reactive protein not log-transformed) | (0.10±0.31) | (0.06±0.10) | (0.07±0.10) | (0.31±0.76) | (0.0214) |
| 16 | Ceruloplasmin (mg/dL) | 25.2±5.6 | 24.6±4.3 | 25.4±6.8 | 25.9±4.7 | 0.6762 |
| 17 | Zinc (μg/dL) | 92.7±11.7 | 93.0±10.3 | 91.8±11.9 | 94.6±14.5 | 0.7191 |
| 18 | 25-hydroxyvitamin D (ng/mL) | 19.3±8.5 | 18.1±7.1 | 20.9±8.9 | 17.9±10.2 | 0.2444 |
| 19 | 1.25-dihydroxyvitamin D of vitamin D2 (ng/mL) | 67.6±20.6 | 63.2±17.2 | 68.5±19.8 | 76.6±28.4 | 0.0951 |
| 20 | Calcium (mEq/L) | 9.1±0.3 | 9.1±0.3 | 9.1±0.3 | 9.0±0.3 | 0.1471 |
| 21 | Magnesium (mg/dL) | 2.23±0.14 | 2.25±0.10 | 2.23±0.15 | 2.19±0.19 | 0.4770 |
| 22 | Sodium (mEq/L) | 138.3±1.6 | 138.0±1.3 | 138.6±1.6 | 138.5±2.0 | 0.2657 |
| 23 | Potassium (mEq/L) | 4.0±0.3 | 4.0±0.2 | 4.0±0.3 | 4.0±0.3 | 0.9555 |
| 24 | Chlorine (mEq/L) | 103.9±1.8 | 103.7±1.6 | 104.0±2.0 | 103.8±2.0 | 0.7125 |
| 25 | Uric acid (mg/dL)^c^ | 5.3±1.5 | 5.1±1.5 | 5.3±1.3 | 5.9±1.8 | 0.1841 |
| 26 | Total protein (g/dL) | 7.2±0.4 | 7.3±0.4 | 7.2±0.3 | 7.2±0.4 | 0.6634 |
| 27 | Albumin (g/dL) | 4.6±0.2 | 4.6±0.2 | 4.6±0.2 | 4.6±0.3 | 0.8502 |
| 28 | Aspartate aminotransferase (IU/L) | 23.2±8.9 | 22.0±6.1 | 23.3±10.9 | 25.9±8.4 | 0.3638 |
| 29 | Alanine aminotransferase (IU/L) | 25.0±19.3 | 23.9±18.0 | 25.7±22.2 | 25.9±13.1 | 0.8996 |
| **30** | **γ-glutamyl transpeptidase (IU/L)** | **32.7±30.9** | **25.5±22.2** | **32.8±24.9** | **51.4±53.8** | **0.0203** |
| 31 | Lactate dehydrogenase (U/L) | 178.6±31.4 | 180.5±35.5 | 174.3±29.0 | 186.6±26.7 | 0.3779 |
| 32 | Alkaline phosphatase (U/L) | 62.4±17.5 | 60.0±17.8 | 62.2±15.9 | 69.1±20.4 | 0.2277 |
| 33 | Total bilirubin (mg/dL) | 0.8±0.3 | 0.8±0.3 | 0.8±0.3 | 0.8±0.3 | 0.8721 |
| 34 | Direct bilirubin (mg/dL) | 0.2±0.1 | 0.2±0.1 | 0.2±0.1 | 0.2±0.1 | 0.6805 |
| 35 | Indirect bilirubin (mg/dL) | 0.6±0.2 | 0.6±0.2 | 0.6±0.3 | 0.6±0.2 | 0.9347 |
| 36 | Choline esterase (U/L) | 321.4±70.2 | 314.6±60.7 | 315.8±69.4 | 356.2±88.2 | 0.1133 |
| 37 | Serum amylase | 80.8±27.6 | 83.9±24.6 | 79.1±26.8 | 77.6±37.5 | 0.6556 |
| 38 | **Serum creatinine (mg/dL)** | **0.74±0.14** | **0.69±0.10** | **0.78±0.16** | **0.73±0.14** | **0.0078** |
| 39 | Blood urea nitrogen (mg/dL) | 13.5±3.2 | 13.9±3.2 | 13.6±3.2 | 12.1±2.9 | 0.1799 |
| 40 | Total cholesterol (mg/dL)^d^ | 207.9±33.6 | 211.9±34.6 | 206.4±34.3 | 201.9±29.5 | 0.5747 |
| 41 | Triglyceride (mg/dL)^d^ | 83.6±49.7 | 70.7±29.2 | 89.3±51.6 | 99.8±75.8 | 0.0896 |
| 42 | High-density lipoprotein cholesterol (mg/dL)^d^ | 67.8±15.7 | 70.5±16.1 | 66.7±14.5 | 64.3±17.9 | 0.3492 |
| 43 | Low-density lipoprotein cholesterol (mg/dL)^d^ | 119.7±31.5 | 122.4±30.1 | 118.5±31.0 | 116.3±37.6 | 0.7715 |
| 44 | Small dense low-density lipoprotein cholesterol (mg/dL)^d^ | 26.9±10.4 | 24.8±8.6 | 27.9±11.2 | 29.3±11.8 | 0.2568 |
| 45 | Phospholipid (mg/dL) | 217.0±26.2 | 218.9±26.7 | 217.0±27.3 | 211.9±21.7 | 0.6791 |
| 46 | Total homocysteine (nmol/mL) | 10.3±4.0 | 9.5±3.9 | 10.7±4.1 | 11.1±3.4 | 0.2961 |
| 47 | Free fatty acid (μEq/L) | 661.6±257.5 | 628.8±220.0 | 675.8±286.1 | 703.4±262.9 | 0.5626 |
| 48 | Gastric pepsinogen I (ng/dL) | 40.6±25.1 | 38.5±20.3 | 42.1±25.4 | 41.4±35.1 | 0.8023 |
| 49 | Gastric pepsinogen I/II ratio | 5.58±1.25 | 5.87±0.98 | 5.47±1.15 | 5.17±1.90 | 0.1243 |
| 50 | Urinary albumin (mg/gCre) (common log-transformed) ^e^ | 1.62±0.36 | 1.53±0.27 | 1.66±0.42 | 1.73±0.32 | 0.1005 |
|  | (Urinary albumin not log-transformed)^e^ | (84.4±255.7) | (40.6±29.1) | (125.3±370.8) | (69.7±51.3) | (0.3130) |

**Signals of association at P-value < 0.05.** ANOVA = analysis of variance, SD = standard deviation.

^a^Two patients in the middle sleep group receiving treatment for hypertension are excluded.

^b^One patient in the better sleep group receiving treatment for diabetes is excluded.

^c^One patient in the middle group receiving treatment for hyperuricemia is excluded.

^d^One patient in the middle sleep group receiving treatment for dyslipidemia is excluded.

^e^One patient in the better sleep group without urine measurement is excluded.

| Variables | (i) Better sleep group (n=39) | (ii) Middle sleep group (n=46) | (iii) Worse sleep group (n=15) |
| --- | --- | --- | --- |
| 1. Systolic blood pressure (mmHg)^a^ | | | |
| Crude values, mean±SD | 103.3±13.2 | 111.2±14.1 | 119.0±14.5 |
| Crude coefficient (95% CI) | Ref. | **7.9 (1.9 – 13.9)** | **15.7 (7.3 – 24.0)** |
| Adjusted coefficient (95% CI) in Model 1 | Ref. | 3.9 (-1.8 – 9.7) | **10.7 (2.8 – 18.5)** |
| Adjusted coefficient (95% CI) in Model 2 | Ref. | 5.0 (-0.4 – 10.3) | **9.5 (2.1 – 17.0)** |
| Adjusted coefficient (95% CI) in Model 3 | Ref. | 5.4 (-0.1 – 10.9) | **8.7 (1.1 – 16.3)** |
| 1. Diastolic blood pressure (mmHg)^a^ | | | |
| Crude values, mean±SD | 63.6±9.9 | 69.5±11.1 | 73.9±11.7 |
| Crude coefficient (95% CI) | Ref. | **5.9 (1.2 – 10.6)** | **10.4 (3.9 – 16.9)** |
| Adjusted coefficient (95% CI) in Model 1 | Ref. | 2.5 (-1.8 – 6.9) | **6.1 (0.2 – 12.1)** |
| Adjusted coefficient (95% CI) in Model 2 | Ref. | 3.5 (-0.8 – 7.8) | 5.8 (-0.2 – 11.8) |
| Adjusted coefficient (95% CI) in Model 3 | Ref. | 4.0 (-0.4 – 8.3) | 5.0 (-1.0 – 11.1) |
| 1. γ-glutamyl transpeptidase (IU/L) | | | |
| Crude values, mean±SD | 25.5±22.2 | 32.8±24.9 | 51.4±53.8 |
| Crude coefficient (95% CI) | Ref. | 7.4 (-5.6 – 20.3) | **25.9 (7.9 – 44.0)** |
| Adjusted coefficient (95% CI) in Model 1 | Ref. | -0.1 (-12.8 – 12.5) | 16.8 (-0.7 – 34.3) |
| Adjusted coefficient (95% CI) in Model 2 | Ref. | 0.8 (-10.2 – 11.8) | 8.1 (-7.4 – 23.5) |
| Adjusted coefficient (95% CI) in Model 3 | Ref. | -0.4 (-11.3 – 10.5) | 4.3 (-11.0 – 19.6) |
| 1. Serum creatinine (mg/dL) | | | |
| Crude values, mean±SD | 0.69±0.10 | 0.78±0.16 | 0.73±0.14 |
| Crude coefficient (95% CI) | Ref. | **0.09 (0.04 – 0.15)** | 0.05 (-0.03 – 0.13) |
| Adjusted coefficient (95% CI) in Model 1 | Ref. | 0.04 (-0.01 – 0.08) | -0.03 (-0.09 – 0.03) |
| Adjusted coefficient (95% CI) in Model 2 | Ref. | 0.04 (-0.004 – 0.09) | -0.02 (-0.08 – 0.05) |
| Adjusted coefficient (95% CI) in Model 3 | Ref. | 0.04 (-0.01 – 0.08) | -0.01 (-0.07 – 0.06) |

**Supplementary Table 3. Results of univariable and multivariable analyses for physical health parameters showing signals of difference**

**Signals of association at P-value < 0.05.** SD = standard deviation, CI = confidence interval. Model 1 is adjusted for age and sex; Model 2 is further adjusted for body mass index, smoking history, and drinking habits; and Model 3 is further adjusted for the 3% oxygen desaturation index. In Model 3, two participants with missing values of 3% oxygen desaturation index are excluded. ^a^Two patients in the middle sleep group receiving treatment for hypertension are excluded.

**Supplementary** **Table 4. Subgroup analysis by sex: Basic characteristics and sleep parameters of the study participants**

| Subgroup: Men | Overall  (n=50) | (i) Better sleep group  (n=13) | (ii) Middle sleep group  (n=27) | (iii) Worse sleep group  (n=10) | P value comparing  the 3 groups |
| --- | --- | --- | --- | --- | --- |
| Age (years), mean±SD | 44.0±8.0 | 37.0±5.4 | 47.1±7.3 | 44.9±7.5 | 0.0003 |
| Body mass index, mean±SD | 22.9±3.9 | 22.8±4.7 | 22.7±2.8 | 23.8±5.6 | 0.7468 |
| Smoking habit, n (%) |  |  |  |  |  |
| No smokers | 32 (64.0) | 10 (76.9) | 17 (63.0) | 5 (50.0) | 0.2266 |
| Past smokers | 9 (18.0) | 0 | 7 (25.9) | 2 (20.0) |  |
| Current smokers | 9 (18.0) | 3 (23.1) | 3 (11.1) | 3 (30.0) |  |
| Drinking habit, n (%) |  |  |  |  |  |
| None | 7 (14.0) | 4 (30.8) | 3 (11.1) | 0 | 0.1573 |
| ≤1 day/week | 18 (36.0) | 4 (30.8) | 11 (40.7) | 3 (30.0) |  |
| 2-5 days/week | 15 (30.0) | 5 (38.5) | 6 (22.2) | 4 (40.0) |  |
| ≥6 days/week | 10 (20.0) | 0 | 7 (25.9) | 3 (30.0) |  |
| 3%ODI (times/hour), mean±SD | 9.5±8.8 | 8.9±8.4 | 8.4±7.0 | 13.2±12.8 |  |
| EEG-based parameters, mean±SD |  |  |  |  |  |
| Total sleep time (min) | 344.8±65.9 | 334.3±60.5 | 338.8±64.0 | 374.6±75.6 | 0.2786 |
| Sleep efficiency (%) | 90.3±4.8 | 93.3±3.6 | 90.2±3.8 | 86.5±6.1 | 0.0019 |
| Sleep latency (min) | 12.0±9.8 | 9.5±9.2 | 11.9±8.2 | 15.4±13.7 | 0.3659 |
| N1% | 10.5±5.3 | 5.9±2.0 | 10.4±3.0 | 17.0±6.8 | <0.0001 |
| N2% | 54.2±7.2 | 48.4±6.9 | 57.6±5.4 | 52.5±6.6 | 0.0002 |
| N3% | 8.6±7.7 | 17.7±5.3 | 4.0±3.6 | 9.2±7.9 | <0.0001 |
| REM% | 26.5±5.0 | 27.9±4.2 | 28.0±4.0 | 20.3±3.6 | <0.0001 |
| WASO (min) | 25.2±17.1 | 15.2±12.5 | 24.3±13.5 | 40.9±20.6 | 0.0007 |
| Arousal index (/hour) | 13.3±4.9 | 10.5±3.3 | 11.9±3.2 | 20.5±3.3 | <0.0001 |
| Sleep stage transitions (/hour) | 8.3±2.2 | 7.2±1.5 | 7.9±1.8 | 10.9±2.1 | <0.0001 |
| Subgroup: Women | Overall  (n=50) | (i) Better sleep group  (n=26) | (ii) Middle sleep group  (n=19) | (iii) Worse sleep group  (n=5) | P value comparing  the 3 groups |
| Age (years), mean±SD | 44.0±9.2 | 44.5±10.1 | 42.5±8.8 | 46.6±6.6 | 0.6292 |
| Body mass index, mean±SD | 21.4±3.4 | 21.1±2.8 | 21.0±2.7 | 24.2±6.7 | 0.1403 |
| Smoking habit, n (%) |  |  |  |  |  |
| No smokers | 43 (86.0) | 24 (92.3) | 15 (79.0) | 4 (80.0) | 0.3850 |
| Past smokers | 5 (10.0) | 2 (7.7) | 2 (10.5) | 1 (20.0) |  |
| Current smokers | 2 (4.0) | 0 | 2 (10.5) | 0 |  |
| Drinking habit, n (%) |  |  |  |  |  |
| None | 22 (44.0) | 12 (46.2) | 8 (42.1) | 2 (40.0) | 0.6131 |
| ≤1 day/week | 17 (34.0) | 8 (30.8) | 8 (42.1) | 1 (20.0) |  |
| 2-5 days/week | 4 (8.0) | 3 (11.5) | 1 (5.3) | 0 |  |
| ≥6 days/week | 7 (14.0) | 3 (11.5) | 2 (10.5) | 2 (40.0) |  |
| 3%ODI (times/hour), mean±SD | 5.2±7.9 | 4.0±6.4 | 5.8±9.9 | 9.3±6.4 | 0.3575 |
| EEG-based parameters, mean±SD |  |  |  |  |  |
| Total sleep time (min) | 352.4±50.1 | 354.2±47.1 | 361.9±48.3 | 307.0±57.8 | 0.0872 |
| Sleep efficiency (%) | 91.3±5.8 | 94.0±2.6 | 90.2±5.0 | 81.2±8.5 | <0.0001 |
| Sleep latency (min) | 10.5±9.7 | 7.1±5.1 | 13.8±12.0 | 15.3±13.9 | 0.0341 |
| N1% | 6.6±3.2 | 5.5±2.7 | 6.8±2.4 | 11.2±4.1 | 0.0005 |
| N2% | 54.3±7.2 | 49.6±4.8 | 59.0±5.8 | 60.8±6.1 | <0.0001 |
| N3% | 11.6±7.4 | 16.6±5.8 | 5.8±4.1 | 8.0±6.9 | <0.0001 |
| REM% | 27.4±4.9 | 28.1±4.3 | 28.4±4.8 | 20.0±1.5 | 0.0010 |
| WASO (min) | 23.5±21.4 | 14.7±7.8 | 26.1±19.3 | 59.4±37.6 | <0.0001 |
| Arousal index (/hour) | 10.3±3.8 | 8.6±2.7 | 10.8±2.9 | 17.3±3.6 | <0.0001 |
| Sleep stage transitions (/hour) | 7.0±1.9 | 6.8±1.6 | 6.6±1.7 | 9.9±2.4 | 0.0010 |

EEG = electroencephalogram, SD = standard deviation, ODI = oxygen desaturation index, REM = rapid eye movement, WASO = wake after sleep onset.

**Supplementary** **Table 5. Subgroup analysis by sex: Results of univariable and multivariable analyses**

| Subgroup: Men | (i) Better sleep group (n=13) | (ii) Middle sleep group (n=27) | (iii) Worse sleep group (n=10) |
| --- | --- | --- | --- |
| 1. Systolic blood pressure (mmHg)^a^ | | | |
| Crude values, mean±SD | 110.2±12.1 | 115.6±11.7 | 120.7±16.7 |
| Crude coefficient (95% CI) | Ref. | 5.4 (-3.4 – 14.2) | 10.5 (-0.4 – 21.5) |
| Adjusted coefficient (95% CI) in Model 1 | Ref. | 1.1 (-9.2 – 11.3) | 7.0 (-4.6 – 18.6) |
| Adjusted coefficient (95% CI) in Model 2 | Ref. | 2.6 (-8.0 – 13.2) | 6.5 (-5.3 – 18.4) |
| Adjusted coefficient (95% CI) in Model 3 | Ref. | 3.6 (-7.1 – 14.3) | 4.7 (-7.2 – 16.7) |
| 1. Diastolic blood pressure (mmHg)^a^ | | | |
| Crude values, mean±SD | 63.6±9.9 | 73.9±8.2 | 75.8±13.2 |
| Crude coefficient (95% CI) | Ref. | 6.3 (-0.4 – 13.0) | 8.2 (-0.1 – 16.4) |
| Adjusted coefficient (95% CI) in Model 1 | Ref. | 1.6 (-5.9 – 9.1) | 4.3 (-4.2 – 12.8) |
| Adjusted coefficient (95% CI) in Model 2 | Ref. | 3.2 (-4.5 – 11.0) | 3.9 (-4.8 – 12.6) |
| Adjusted coefficient (95% CI) in Model 3 | Ref. | 4.5 (-3.3 – 12.2) | 2.8 (-5.8 – 11.5) |
| 1. γ-glutamyl transpeptidase (IU/L) | | | |
| Crude values, mean±SD | 40.3±32.0 | 36.7±25.2 | 62.6±63.8 |
| Crude coefficient (95% CI) | Ref. | -3.6 (-28.9 – 21.7) | 22.3 (-9.3 – 53.9) |
| Adjusted coefficient (95% CI) in Model 1 | Ref. | -24.2 (-52.3 – 3.9) | 6.2 (-25.7 – 38.0) |
| Adjusted coefficient (95% CI) in Model 2 | Ref. | -14.8 (-37.4 – 7.8) | 0.1 (-25.0 – 25.3) |
| Adjusted coefficient (95% CI) in Model 3 | Ref. | -17.6 (-40.1 – 5.0) | -7.7 (-32.8 – 17.4) |
| 1. Serum creatinine (mg/dL) | | | |
| Crude values, mean±SD | 0.78±0.06 | 0.87±0.12 | 0.80±0.11 |
| Crude coefficient (95% CI) | Ref. | **0.09 (0.02 – 0.16)** | 0.02 (-0.07 – 0.11) |
| Adjusted coefficient (95% CI) in Model 1 | Ref. | 0.06 (-0.02 – 0.14) | -0.01 (-0.10 – 0.09) |
| Adjusted coefficient (95% CI) in Model 2 | Ref. | 0.06 (-0.03 – 0.16) | 0.001 (-0.10 – 0.11) |
| Adjusted coefficient (95% CI) in Model 3 | Ref. | 0.05 (-0.05 – 0.15) | 0.01 (-0.10 – 0.12) |

**Signals of association at P-value < 0.05**. SD = standard deviation, CI = confidence interval. Model 1 is adjusted for age and sex; Model 2 is further adjusted for body mass index, smoking history, and drinking habits; and Model 3 is further adjusted for the 3% oxygen desaturation index. In Model 3, two participants with missing values of 3% oxygen desaturation index are excluded. ^a^One patient in the middle sleep group receiving treatment for hypertension is excluded.

| Subgroup: Women | (i) Better sleep group (n=26) | (ii) Middle sleep group (n=19) | (iii) Worse sleep group (n=5) |
| --- | --- | --- | --- |
| 1. Systolic blood pressure (mmHg)^a^ | | | |
| Crude values, mean±SD | 99.9±12.6 | 104.9±15.3 | 115.6±9.4 |
| Crude coefficient (95% CI) | Ref. | 5.0 (-3.3 – 13.3) | **15.6 (2.4 – 28.8)** |
| Adjusted coefficient (95% CI) in Model 1 | Ref. | 6.1 (-1.7 – 13.9) | **14.5 (2.1 – 27.0)** |
| Adjusted coefficient (95% CI) in Model 2 | Ref. | **7.6 (0.2 – 14.9)** | 9.9 (-2.5 – 22.3) |
| Adjusted coefficient (95% CI) in Model 3 | Ref. | **7.6 (0.2 – 15.0)** | 9.3 (-3.6 – 22.1) |
| 1. Diastolic blood pressure (mmHg)^a^ | | | |
| Crude values, mean±SD | 61.6±9.7 | 63.1±11.8 | 70.3±8.1 |
| Crude coefficient (95% CI) | Ref. | 1.6 (-4.9 – 8.0) | 8.7 (-1.5 – 19.0) |
| Adjusted coefficient (95% CI) in Model 1 | Ref. | 2.3 (-4.0 – 8.5) | 8.0 (-1.8 – 17.9) |
| Adjusted coefficient (95% CI) in Model 2 | Ref. | 3.9 (-2.3 – 10.1) | 7.4 (-3.0 – 17.8) |
| Adjusted coefficient (95% CI) in Model 3 | Ref. | 3.9 (-2.3 – 10.2) | 6.9 (-3.9 – 17.7) |
| 1. γ-glutamyl transpeptidase (IU/L) | | | |
| Crude values, mean±SD | 18.0±9.3 | 27.3±24.0 | 29.0±4.5 |
| Crude coefficient (95% CI) | Ref. | 9.3 (-0.7 – 19.2) | 11.0 (-5.2 – 27.1) |
| Adjusted coefficient (95% CI) in Model 1 | Ref. | **10.5 (0.9 – 20.0)** | 9.7 (-5.7 – 25.1) |
| Adjusted coefficient (95% CI) in Model 2 | Ref. | 10.4 (-0.05 – 20.8) | 10.0 (-7.7 – 27.6) |
| Adjusted coefficient (95% CI) in Model 3 | Ref. | 10.6 (-0.004 – 21.2) | 10.2 (-7.7 – 28.0) |
| 1. Serum creatinine (mg/dL) | | | |
| Crude values, mean±SD | 0.64±0.08 | 0.65±0.10 | 0.60±0.09 |
| Crude coefficient (95% CI) | Ref. | 0.01 (-0.04 – 0.06) | -0.04 (-0.12 – 0.05) |
| Adjusted coefficient (95% CI) in Model 1 | Ref. | 0.01 (-0.04 – 0.06) | -0.04 (-0.13 – 0.04) |
| Adjusted coefficient (95% CI) in Model 2 | Ref. | 0.02 (-0.04 – 0.08) | -0.03 (-0.13 – 0.07) |
| Adjusted coefficient (95% CI) in Model 3 | Ref. | 0.03 (-0.03 – 0.08) | -0.02 (-0.12 – 0.07) |

**Signals of association at P-value < 0.05.** SD = standard deviation, CI = confidence interval. Model 1 is adjusted for age and sex; Model 2 is further adjusted for body mass index, smoking history, and drinking habits; and Model 3 is further adjusted for the 3% oxygen desaturation index. ^a^One patient in the middle sleep group receiving treatment for hypertension is excluded.

**Supplementary Figure 4. Distribution of people according to the Athens Insomnia Scale and electroencephalogram-based sleep clusters**


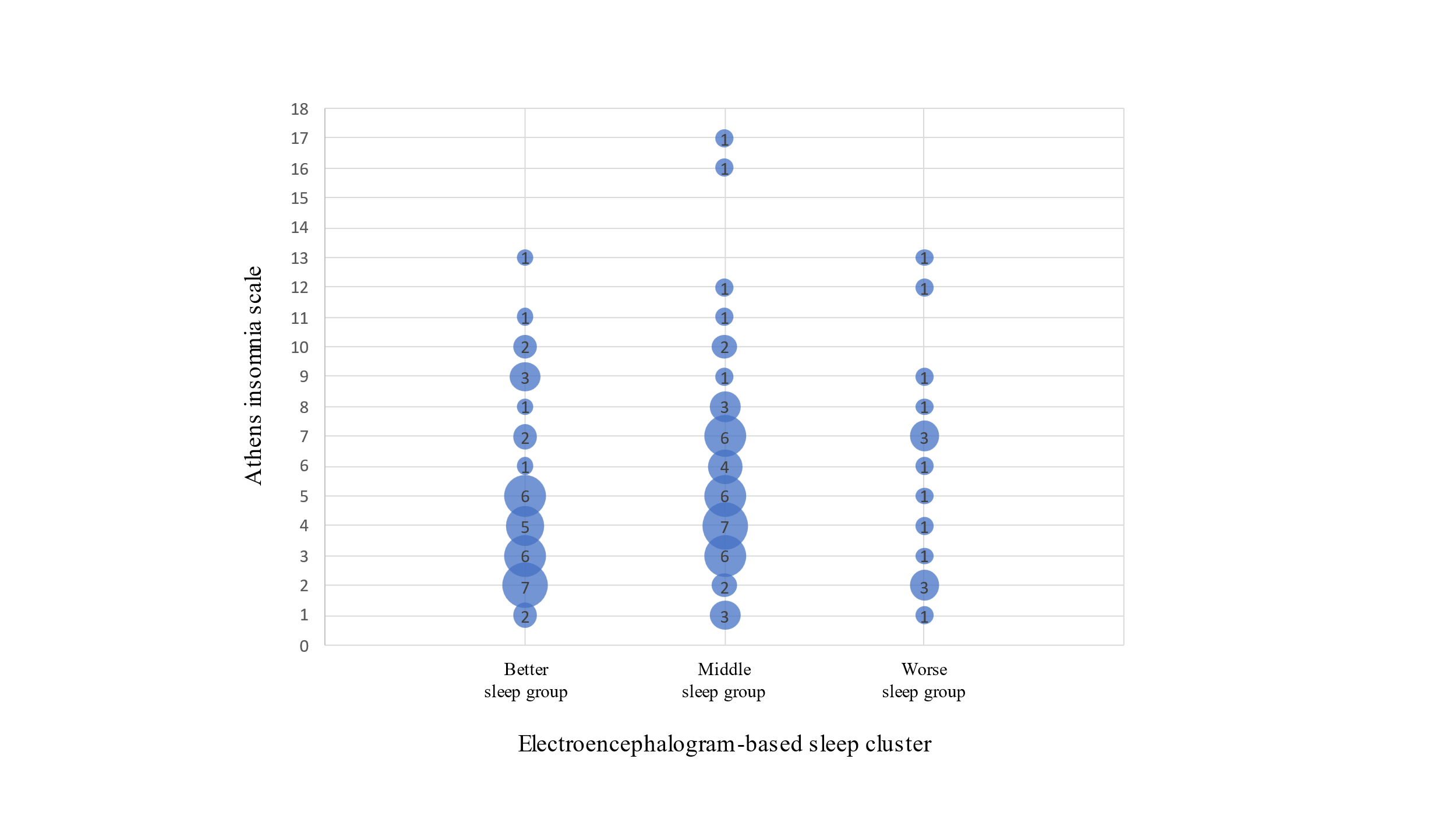


Note: Four participants with missing values of Athens Insomnia Scale are excluded.

**Supplementary Table 6. Sensitivity analyses for systolic blood pressure**

| 1. Sensitivity analysis excluding one patient using sleeping medication | | | |
| --- | --- | --- | --- |
|  | (i) Better sleep group (n=39) | (ii) Middle sleep group (n=43^a^) | (iii) Worse sleep group (n=15) |
| Crude values (mmHg), mean±SD | 103.3±13.2 | 111.2±14.3 | 119.0±14.5 |
| Crude coefficient (95% CI) | Ref. | **7.9 (1.8 – 14.0)** | **15.7 (7.3 – 24.1)** |
| Adjusted coefficient (95% CI) in Model 1 | Ref. | 4.0 (-1.7 – 9.8) | **10.6 (2.7 – 18.4)** |
| Adjusted coefficient (95% CI) in Model 2 | Ref. | 5.2 (-0.2 – 10.6) | **9.6 (2.1 – 17.1)** |
| Adjusted coefficient (95% CI) in Model 3 | Ref. | **5.6 (0.1 – 11.1)** | **8.8 (1.1 – 16.4)** |
| 1. Sensitivity analysis adjusting for the average 3% oxygen desaturation index of two nights (in Model 3) instead of the worse value in the main analysis | | | |
|  | (i) Better sleep group (n=39) | (ii) Middle sleep group (n=44^a^) | (iii) Worse sleep group (n=15) |
| Crude values (mmHg), mean±SD | 103.3±13.2 | 111.2±14.1 | 119.0±14.5 |
| Crude coefficient (95% CI) | Ref. | **7.9 (1.9 – 13.9)** | **15.7 (7.3 – 24.0)** |
| Adjusted coefficient (95% CI) in Model 1 | Ref. | 3.9 (-1.8 – 9.7) | **10.7 (2.8 – 18.5)** |
| Adjusted coefficient (95% CI) in Model 2 | Ref. | 5.0 (-0.4 – 10.3) | **9.5 (2.1 – 17.0)** |
| Adjusted coefficient (95% CI) in Model 3 | Ref. | **5.5 (0.01 – 10.9)** | **8.5 (0.8 – 16.1)** |

SD = standard deviation, CI = confidence interval. Model 1 is adjusted for age and sex; Model 2 is further adjusted for body mass index, smoking history, and drinking habits; and Model 3 is further adjusted for the 3% oxygen desaturation index. In Model 3, two participants with missing values of 3% oxygen desaturation index are excluded. ^a^Two patients in the middle sleep group receiving treatment for hypertension are excluded.

**Supplementary Table 7. Correlation coefficients between each electroencephalogram-based sleep parameter and physical health parameter**

|  | Total sleep time | Sleep efficiency | Sleep latency | N1% | N2% | N3% | REM% | WASO | Arousal index | Sleep stage transitions |
| --- | --- | --- | --- | --- | --- | --- | --- | --- | --- | --- |
| Systolic blood pressure (mmHg)^a^ | -0.073 | -0.187 | 0.052 | **0.388** | 0.160 | **-0.265** | **-0.206** | **0.204** | **0.261** | **0.239** |
| Diastolic blood pressure (mmHg)^a^ | 0.008 | -0.120 | 0.037 | **0.396** | 0.102 | **-0.241** | -0.161 | 0.134 | **0.258** | **0.247** |
| Fasting blood glucose (mg/dL)^b^ | 0.014 | **-0.341** | 0.161 | **0.313** | 0.073 | -0.182 | -0.138 | **0.361** | **0.235** | 0.170 |
| HbA1c (%)^b^ | -0.044 | **-0.246** | -0.013 | 0.055 | 0.083 | -0.094 | -0.038 | **0.301** | 0.096 | 0.140 |
| Glycoalbumin (%)^b^ | 0.115 | 0.097 | -0.179 | -0.140 | 0.060 | 0.067 | -0.057 | -0.025 | -0.062 | 0.022 |
| 1,5-anhydro-D-glucitol (μg/mL)^b^ | 0.003 | 0.023 | 0.038 | 0.075 | -0.101 | -0.012 | 0.095 | -0.099 | 0.139 | 0.179 |
| Hemoglobin (g/dL) | -0.157 | -0.115 | 0.062 | **0.363** | -0.028 | -0.154 | -0.069 | 0.039 | **0.227** | **0.232** |
| White blood cell count (/μL) | -0.095 | -0.148 | 0.151 | -0.042 | -0.008 | 0.053 | -0.014 | 0.068 | -0.028 | -0.166 |
| Platelet count (10^4^/μL) | **-0.216** | -0.177 | 0.078 | -0.049 | 0.042 | 0.040 | -0.065 | 0.129 | -0.017 | -0.058 |
| Total iron binding capacity (μg/dL) | 0.050 | -0.056 | 0.016 | 0.148 | 0.090 | -0.160 | -0.038 | 0.106 | 0.063 | 0.007 |
| Unsaturated iron binding capacity (μg/dL) | 0.012 | -0.088 | 0.004 | 0.066 | 0.002 | -0.048 | -0.003 | 0.133 | 0.093 | 0.024 |
| Ferritin (ng/mL) | -0.076 | -0.031 | 0.044 | 0.104 | 0.012 | -0.046 | -0.050 | -0.024 | 0.167 | **0.298** |
| Serum iron (μg/dL) | 0.042 | 0.080 | 0.013 | 0.073 | 0.108 | -0.118 | -0.042 | -0.094 | -0.079 | -0.031 |
| Serum copper (μg/dL) | -0.026 | 0.021 | -0.035 | -0.043 | **0.234** | -0.134 | -0.081 | 0.005 | -0.067 | 0.040 |
| Log-transformed C-reactive protein (mg/dL) | -0.033 | -0.165 | **0.207** | 0.130 | 0.113 | -0.125 | -0.081 | 0.129 | -0.043 | -0.028 |
| Ceruloplasmin (mg/dL) | -0.009 | -0.018 | -0.024 | -0.052 | **0.233** | -0.134 | -0.072 | 0.050 | -0.053 | 0.016 |
| Zinc (μg/dL) | 0.111 | **0.220** | -0.124 | 0.004 | -0.109 | 0.035 | 0.092 | -0.172 | 0.102 | 0.118 |
| 25-hydroxyvitamin D (ng/mL) | 0.032 | 0.162 | -0.085 | 0.013 | 0.003 | -0.103 | 0.144 | -0.182 | -0.011 | 0.069 |
| 1.25-dihydroxyvitamin D of vitamin D2 (ng/mL) | -0.035 | -0.042 | -0.066 | 0.158 | 0.093 | -0.166 | -0.036 | 0.079 | 0.118 | 0.150 |
| Calcium (mEq/L) | 0.077 | 0.151 | -0.096 | -0.139 | -0.063 | 0.130 | 0.028 | -0.110 | -0.007 | 0.095 |
| Magnesium (mg/dL) | 0.092 | 0.113 | -0.168 | -0.017 | -0.013 | 0.088 | -0.119 | 0.033 | -0.012 | 0.083 |
| Sodium (mEq/L) | -0.061 | 0.063 | -0.047 | 0.159 | 0.056 | -0.126 | -0.042 | -0.104 | 0.132 | **0.248** |
| Potassium (mEq/L) | -0.007 | 0.127 | -0.181 | 0.082 | 0.078 | -0.196 | 0.116 | -0.077 | 0.089 | 0.136 |
| Chlorine (mEq/L) | 0.015 | 0.025 | 0.041 | 0.034 | 0.055 | -0.125 | 0.052 | -0.066 | 0.034 | 0.034 |
| Uric acid (mg/dL)^c^ | 0.061 | -0.126 | **0.248** | **0.266** | -0.095 | -0.032 | -0.089 | 0.059 | 0.191 | 0.088 |
| Total protein (g/dL) | 0.073 | 0.145 | -0.143 | -0.096 | -0.084 | 0.174 | -0.041 | -0.049 | -0.003 | 0.048 |
| Albumin (g/dL) | 0.187 | 0.059 | -0.044 | 0.036 | -0.135 | 0.100 | 0.002 | 0.012 | 0.106 | 0.051 |
| Aspartate aminotransferase (IU/L) | -0.032 | -0.072 | 0.083 | 0.068 | 0.162 | -0.114 | -0.130 | 0.046 | 0.151 | 0.016 |
| Alanine aminotransferase (IU/L) | 0.011 | -0.069 | 0.183 | -0.006 | 0.095 | -0.049 | -0.061 | 0.011 | 0.005 | -0.095 |
| γ-glutamyl transpeptidase (IU/L) | 0.086 | -0.093 | 0.149 | **0.447** | -0.001 | -0.149 | **-0.208** | 0.087 | **0.309** | **0.231** |
| Lactate dehydrogenase (U/L) | -0.128 | 0.008 | -0.067 | 0.109 | 0.029 | -0.036 | -0.100 | -0.006 | 0.091 | 0.130 |
| Alkaline phosphatase (U/L) | -0.137 | -0.193 | 0.100 | 0.189 | -0.001 | -0.040 | -0.120 | 0.139 | 0.172 | **0.274** |
| Total bilirubin (mg/dL) | -0.109 | 0.071 | -0.087 | 0.000 | -0.053 | 0.042 | 0.019 | -0.085 | 0.018 | 0.060 |
| Direct bilirubin (mg/dL) | -0.024 | 0.075 | -0.083 | 0.009 | -0.059 | 0.054 | 0.001 | -0.068 | 0.042 | 0.052 |
| Indirect bilirubin (mg/dL) | -0.134 | 0.065 | -0.084 | -0.003 | -0.048 | 0.036 | 0.024 | -0.086 | 0.009 | 0.060 |
| Choline esterase (U/L) | -0.128 | -0.056 | 0.034 | 0.140 | 0.011 | -0.014 | -0.133 | 0.027 | 0.129 | 0.119 |
| Serum amylase | 0.104 | 0.070 | 0.024 | -0.047 | -0.031 | 0.083 | -0.029 | -0.070 | -0.031 | 0.037 |
| Serum creatinine (mg/dL) | -0.063 | -0.086 | 0.123 | **0.320** | 0.063 | **-0.271** | 0.020 | 0.033 | 0.182 | 0.166 |
| Blood urea nitrogen (mg/dL） | 0.103 | **0.209** | -0.152 | -0.094 | -0.108 | 0.062 | 0.159 | -0.124 | -0.170 | 0.010 |
| Total cholesterol (mg/dL)^d^ | -0.124 | 0.067 | -0.028 | 0.044 | -0.097 | 0.048 | 0.016 | -0.086 | -0.021 | 0.084 |
| Triglyceride (mg/dL)^d^ | 0.037 | -0.074 | 0.175 | **0.377** | 0.088 | **-0.266** | -0.101 | 0.047 | 0.211 | 0.164 |
| High-density lipoprotein cholesterol (mg/dL)^d^ | -0.056 | 0.114 | -0.197 | -0.135 | 0.023 | 0.036 | 0.055 | -0.087 | -0.132 | -0.085 |
| Low-density lipoprotein cholesterol (mg/dL)^d^ | -0.113 | 0.033 | 0.018 | 0.073 | -0.131 | 0.065 | 0.007 | -0.056 | 0.023 | 0.127 |
| Small dense low-density lipoprotein cholesterol (mg/dL)^d^ | -0.070 | -0.022 | 0.076 | **0.287** | 0.018 | -0.164 | -0.074 | 0.001 | 0.175 | **0.246** |
| Phospholipid (mg/dL) | -0.093 | 0.103 | -0.010 | 0.116 | -0.078 | -0.068 | 0.097 | -0.145 | -0.002 | 0.053 |
| Total homocysteine (nmol/mL) | **-0.268** | **-0.235** | 0.186 | 0.184 | 0.047 | -0.057 | -0.157 | 0.090 | 0.189 | 0.149 |
| Free fatty acid (μEq/L) | -0.053 | -0.067 | -0.013 | -0.027 | 0.068 | -0.008 | -0.055 | 0.078 | -0.023 | -0.050 |
| Gastric pepsinogen I (ng/dL) | -0.009 | 0.147 | -0.107 | 0.115 | -0.004 | -0.081 | 0.019 | -0.148 | 0.054 | 0.151 |
| Gastric pepsinogen I/II ratio | 0.040 | 0.148 | **-0.220** | **-0.250** | -0.014 | **0.248** | -0.116 | -0.102 | -0.148 | 0.079 |
| Urinary albumin (mg/gCre) | 0.048 | -0.088 | 0.063 | 0.193 | 0.011 | -0.164 | 0.063 | 0.109 | 0.013 | 0.031 |

**Signals of correlation at P-value <0.05.**

^a^Two patients in the middle sleep group receiving treatment for hypertension are excluded.

^b^One patient in the better sleep group receiving treatment for diabetes is excluded.

^c^One patient in the middle group receiving treatment for hyperuricemia is excluded.

^d^One patient in the middle sleep group receiving treatment for dyslipidemia is excluded.

^e^One patient in the better sleep group without urine measurement is excluded.

**Supplementary Table 8. Results of univariable and multivariable analyses in the association between each electroencephalogram-based sleep parameter and physical health parameters showing signals of correlation**

| EEG-based sleep parameter (exposure in the model) | | Total sleep time (min) | | Sleep efficiency (%) | | |
| --- | --- | --- | --- | --- | --- | --- |
| Physical health parameter (outcome in the model) | | Platelet count (10^4^/μL) | **Total homocysteine (nmol/mL)** | **Fasting blood glucose (mg/dL)^b^** | HbA1c (%)^b^ | **Zinc (μg/dL)** |
| Crude coefficient (95% CI) per 1SD increase | | **-1.2**  **(-2.3 – -0.1)** | **-1.1**  **(-1.8 – -0.3)** | **-3.1**  **(-4.8 – -1.4)** | **-0.07**  **(-0.13 – -0.01)** | **2.6**  **(0.3 – 4.8)** |
| Adjusted coefficient (95% CI) per 1SD increase in Model 1 | | **-1.3**  **(-2.4 – -0.2)** | **-0.9**  **(-1.6 – -0.2)** | **-2.7**  **(-4.3 – -1.1)** | **-0.06**  **(-0.12 – -0.01)** | **2.8**  **(0.5 – 5.1)** |
| Adjusted coefficient (95% CI) per 1SD increase in Model 2 | | -0.8  (-1.8 – 0.2) | **-0.7**  **(-1.4 – -0.02)** | **-2.0**  **(-3.5 – -0.4)** | -0.05  (-0.11 – 0.002) | **3.2**  **(0.8 – 5.7)** |
| Adjusted coefficient (95% CI) per 1SD increase in Model 3 | | -0.7  (-1.8 – 0.3) | **-0.8**  **(-1.5 – -0.1)** | **-1.9**  **(-3.5 – -0.3)** | -0.05  (-0.11 – 0.004) | **3.6**  **(1.2 – 5.9)** |
| Sleep efficiency (%) | | Sleep latency (min) | | | N1% | |
| **Blood urea nitrogen (mg/dL)** | Total homocysteine (nmol/mL) | Log-transformed C-reactive protein (mg/dL) | Uric acid (mg/dL)^c^ | Gastric pepsinogen I/II ratio | Systolic blood pressure (mmHg)^a^ | Diastolic blood pressure (mmHg)^a^ |
| **0.7**  **(0.04 – 1.3)** | **-0.9**  **(-1.7 – -0.2)** | **0.10**  **(0.01 – 0.20)** | **0.4**  **(0.1 – 0.7)** | **-0.27**  **(-0.52 – -0.03)** | **5.7**  **(2.9 – 8.4)** | **4.4**  **(2.4 – 6.5)** |
| **0.8**  **(0.2 – 1.4)** | **-0.7**  **(-1.4 – -0.01)** | **0.11**  **(0.01 -0.20)** | **0.3**  **(0.04 – 0.56)** | **-0.28**  **(-0.53 – -0.04)** | 2.8  (-0.1 – 5.7) | 2.0  (-0.2 – 4.2) |
| **0.7**  **(0.1 – 1.4)** | -0.4  (-1.1 – 0.4) | 0.04  (-0.04 – 0.13) | 0.2  (-0.1 – 0.5) | -0.25  (-0.51 – 0.02) | 2.1  (-0.8 – 4.9) | 1.8  (-0.4 – 4.1) |
| **0.7**  **(0.1 – 1.4)** | -0.4  (-1.1 – 0.3) | 0.03  (-0.06 – 0.12) | 0.2  (-0.04 – 0.5) | -0.23  (-0.5 – 0.04) | 1.6  (-1.4 – 4.5) | 1.4  (-0.9 – 3.7) |
| N1% | | | | | | |
| Fasting blood glucose (mg/dL)^b^ | Hemoglobin (g/dL) | Uric acid (mg/dL)^c^ | **γ-glutamyl transpeptidase (IU/L)** | Serum creatinine (mg/dL) | **Triglyceride (mg/dL)^d^** | Small dense low-density lipoprotein cholesterol (mg/dL)^d^ |
| **2.9**  **(1.1 – 4.6)** | **0.5**  **(0.3 – 0.8)** | **0.4**  **(0.1 – 0.7)** | **13.8**  **(8.3 – 19.4)** | **0.04**  **(0.02 – 0.07)** | **18.6**  **(9.4 – 27.9)** | **3.0**  **(1.0 – 5.0)** |
| **2.0**  **(0.02 – 3.9)** | 0.04  (-0.2 – 0.3) | 0.2  (-0.1 – 0.5) | **10.0**  **(3.8 – 16.1)** | -0.003  (-0.03 – 0.02) | **14.0**  **(3.6 – 24.3)** | 1.7  (-0.5 – 4.0) |
| 1.0  (-0.8 – 2.9) | -0.003  (-0.2 – 0.2) | 0.1  (-0.2 – 0.4) | **7.3**  **(1.8 – 12.8)** | 0.002  (-0.02 – 0.03) | **13.4**  **(3.5 – 23.4)** | 1.2  (-1.1 – 3.5) |
| 1.3  (-0.6 – 3.2) | -0.01  (-0.2 – 0.2) | 0.1  (-0.2 – 0.4) | **6.4**  **(0.8 – 11.9)** | 0.01  (-0.02 – 0.03) | **13.7**  **(3.4 – 24.1)** | 1.2  (-1.2 – 3.6) |
| N1% | N2% | | N3% | | | |
| Gastric pepsinogen I/II ratio | Serum copper (μg/dL) | Ceruloplasmin (mg/dL) | Systolic blood pressure (mmHg)^a^ | Diastolic blood pressure (mmHg)^a^ | Serum creatinine (mg/dL) | Triglyceride (mg/dL)^d^ |
| **-0.31**  **(-0.55 – -0.07)** | **5.4**  **(0.9 – 9.9)** | **1.3**  **(0.2 – 2.4)** | **-3.9**  **(-6.8 – -1.0)** | **-2.7**  **(-5.0 – -0.5)** | **-0.04**  **(-0.07 – -0.01)** | **-14.4**  **(-24.2 – -4.6)** |
| **-0.33**  **(-0.60 – -0.05)** | **4.8**  **(0.5 – 9.0)** | **1.2**  **(0.1 – 2.2)** | -1.9  (-4.6 – 0.8) | -1.0  (-3.1 – 1.0) | -0.01  (-0.03 – 0.01) | **-10.2**  **(-20.2 – -0.2)** |
| -0.27  (-0.57 – 0.02) | **4.5**  **(0.2 – 8.9)** | **1.1**  **(0.1 – 2.2)** | -1.8  (-4.4 – 0.7) | -1.1  (-3.1 – 0.9) | -0.02  (-0.04 – 0.004) | -9.1  (-18.3 – 0.05) |
| -0.27  (-0.58 – 0.03) | 4.5  (-0.03 – 9.1) | 1.1  (-0.03 – 2.2) | -1.9  (-4.5 – 0.6) | -1.3  (-3.3 – 0.8) | -0.02  (-0.04 – 0.004) | -8.9  (-18.2 – 0.4) |
| N3% | REM% | | Wake after sleep onset (min) | | | Arousal index |
| Gastric pepsinogen I/II ratio | Systolic blood pressure (mmHg)^a^ | γ-glutamyl transpeptidase (IU/L) | Systolic blood pressure (mmHg)^a^ | **Fasting blood glucose (mg/dL)^b^** | **HbA1c (%)^b^** | Systolic blood pressure (mmHg)^a^ |
| **0.31**  **(0.07 – 0.55)** | **-3.1**  **(-6.2 – -0.1)** | **-6.4**  **(-12.5 – -0.4)** | **3.0**  **(0.1 – 5.9)** | **3.3**  **(1.6 – 5.0)** | **0.09**  **(0.03 – 0.14)** | **3.8**  **(1.0 – 6.7)** |
| **0.30**  **(0.04 – 0.55)** | -2.4  (-5.1 – 0.3) | -5.0  (-10.6 – 0.7) | 2.2  (-0.4 – 4.8) | **2.9**  **(1.2 – 4.5)** | **0.07**  **(0.02 – 0.13)** | 1.4  (-1.4 – 4.2) |
| 0.25  (-0.02 – 0.51) | -1.4  (-3.9 – 1.2) | -2.8  (-7.6 – 2.1) | 1.2  (-1.3 – 3.7) | **2.5**  **(0.95 – 4.0)** | **0.07**  **(0.02 – 0.13)** | 1.3  (-1.3 – 4.0) |
| 0.24  (-0.03 – 0.51) | -1.3  (-4.0 – 1.3) | -2.6  (-7.5 – 2.3) | 1.2  (-1.3 – 3.7) | **2.4**  **(0.9 – 3.9)** | **0.07**  **(0.02 – 0.13)** | 0.9  (-1.7 – 3.6) |
| Arousal index (/hour) | | | | Sleep stage transitions (/hour) | | |
| Diastolic blood pressure (mmHg)^a^ | Fasting blood glucose (mg/dL)^b^ | Hemoglobin (g/dL) | γ-glutamyl transpeptidase (IU/L) | Systolic blood pressure (mmHg)^a^ | Diastolic blood pressure (mmHg)^a^ | Hemoglobin (g/dL) |
| **2.9**  **(0.7 – 5.1)** | **2.1**  **(0.4 – 3.9)** | **0.3**  **(0.05 – 0.6)** | **9.6**  **(3.7 – 15.4)** | **3.8**  **(1.0 – 6.7)** | **2.9**  **(0.7 – 5.1)** | **0.3**  **(0.05 – 0.6)** |
| 0.9  (-1.2 – 3.0) | 1.4  (-0.4 – 3.3) | -0.1  (-0.3 – 0.1) | 5.9  (-0.02 – 11.9) | 1.4  (-1.4 – 4.2) | 0.9  (-1.2 – 3.0) | -0.1  (-0.3 – 0.1) |
| 0.8  (-1.3 – 2.9) | 1.4  (-0.2 – 3.1) | -0.1  (-0.3 – 0.1) | 4.2  (-0.9 – 9.3) | 1.3  (-1.3 – 4.0) | 0.8  (-1.3 – 2.9) | -0.1  (-0.3 – 0.1) |
| 0.4  (-1.7 – 2.5) | 1.6  (-0.03 – 3.2) | -0.1  (-0.3 – 0.1) | 3.7  (-1.4 – 8.7) | 0.9  (-1.7 – 3.6) | 0.4  (-1.7 – 2.5) | -0.1  (-0.3 – 0.1) |
| Sleep stage transitions (/hour) | | | | |  |  |
| **Ferritin (ng/mL)** | Sodium (mEq/L) | γ-glutamyl transpeptidase (IU/L) | Alkaline phosphatase (U/L) | Small dense low-density lipoprotein cholesterol (mg/dL)^d^ |  |  |
| **28.7**  **(10.3 – 47.0)** | **0.4**  **(0.1 – 0.7)** | **7.1**  **(1.1 – 13.2)** | **4.8**  **(1.4 – 8.1)** | **2.6**  **(0.5 – 4.6)** |  |  |
| 14.1  (-4.3 – 32.5) | 0.1  (-0.2 – 0.4) | 1.9  (-4.4 – 8.2) | 1.9  (-1.6 – 5.4) | 1.3  (-0.9 – 3.5) |  |  |
| **20.4**  **(2.3 – 38.6)** | 0.2  (-0.1 – 0.5) | 3.3  (-2.0 – 8.7) | 2.2  (-1.4 – 5.8) | 1.7  (-0.5 – 3.8) |  |  |
| **19.3**  **(1.2 – 37.3)** | 0.2  (-0.1 – 0.5) | 2.3  (-3.0 – 7.6) | 1.9  (-1.7 – 5.5) | 1.7  (-0.6 – 3.9) |  |  |

**Signals of association at P-value <0.05.**

Model 1 is adjusted for age and sex; Model 2 is further adjusted for body mass index, smoking history, and drinking habits; and Model 3 is further adjusted for the 3% oxygen desaturation index. In Model 3, two participants with missing values of 3% oxygen desaturation index are excluded.

^a^Two patients in the middle sleep group receiving treatment for hypertension are excluded.

^b^One patient in the better sleep group receiving treatment for diabetes is excluded.

^c^One patient in the middle group receiving treatment for hyperuricemia is excluded.

^d^One patient in the middle sleep group receiving treatment for dyslipidemia is excluded.
